# Gradual Coordination and Reversible P–P Bond Activation of a P_3_‐Unit with Transition Metal Carbonyls

**DOI:** 10.1002/advs.202306805

**Published:** 2023-12-31

**Authors:** Roman Franz, Dalma Gál, Clemens Bruhn, Zsolt Kelemen, Rudolf Pietschnig

**Affiliations:** ^1^ Institute for Chemistry and CINSaT University of Kassel Heinrich‐Plett‐Straße 40 34132 Kassel Germany; ^2^ Department of Inorganic and Analytical Chemistry Budapest University of Technology and Economics Műegyetem Rkp 3. Budapest 1111 Hungary

**Keywords:** bond activation, ferrocene, metal carbonyl, multinuclear NMR, phosphorus

## Abstract

Coordination of a stereochemically defined P_3_‐chain to a series of transition metal carbonyls [M(CO)_x_]^z−^ (M = Mn (x = 5, z = 1), Fe (x = 4, z = 2) or Co (x = 4, z = 1)) is explored using a [3]ferrocenophane scaffold. A gradual transition from η^1^‐, η^2^‐ to η^3^‐coordination is observed where in the η^2^‐mode the terminal positions of the phosphorus chain are bridged. With an excess of cobalt carbonyl successive P–P bond activation and gradual separation of the central phosphorus atoms from the phosphorus chain has been observed. This process is reversible and with suitable reagents such as methyl lithium, the P_3_‐unit is regenerated in stereospecific manner. The bonding situation and steps of the gradual P–P bond activation are investigated by DFT calculations as well as experimental methods (e.g., NMR spectroscopy, X‐ray crystallography).

## Graphical abstract

Reversible P–P bond activation of a stereochemically preorganized P_3_‐unit is observed with cobalt carbonyl. The process involves gradual fragmentation into P_2_‐ and P_1_‐units which can be reverted to the initial P_3_‐chain with nucleophiles. Coordination of the P_3_‐unit to several transition metal carbonyls is further explored featuring preferential coordination at the terminal positions and variable coordination at the central phosphorus atom.

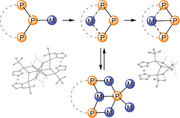

## 1 Introduction

Bond activation under mild conditions is a rewarding yet challenging topic in synthetic chemistry. Activation of P–P bonds in the coordination sphere of transition metals is particularly attractive, paving the way to sustainable production of organophosphorus compounds which play a pivotal role for application in catalysis, additives in consumer products, as medication or luminescent molecular materials to name just a few. While initial groundbreaking investigations focused on direct activation of P_4_,^[^ ^1^ ^]^ in recent years selective P–P bond activation in substituted prochiral oligophosphorus compounds moved into the focus of research targeting transition metal complexes with bisphosphanide ligands with the ultimate goal of stereochemical control.^[^ ^2^ ^]^

Transition metal complexes of formally disubstituted PR_2_ ligands are very multi‐faceted in terms of metal‐phosphorus interaction and electron transfer covering a range of charged phosphanide versus phosphenium species or neutral radical species on the side of the phosphorus fragments.^[^ ^3^ ^]^ The coordination of formally cationic diaminophosphenium ligands [(>N)_2_P]^+^ has been particularly studied, owing to their close resemblance to (hetero‐)carbene species.^[^ ^4^ ^]^ Recently phosphenium transition metal complexes were demonstrated undergoing hydrogen activation and hydrogen transfer to substrates.^[^ ^5^ ^]^ We were intrigued by the question how formal replacement of the stabilizing amino units in [(>N)_2_P]^+^ by isolobal phosphanyl units would modify the reactivity of the central phosphorus atom in the underlying cationic triphosphane fragment [(>P)_2_P]^+^.

**Figure 1:**
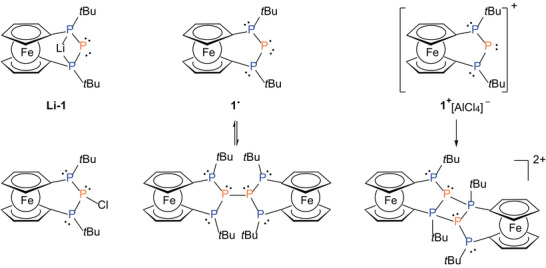
Varieties of the PPP‐[3]ferrocenophane scaffold (anionic (top left), cationic (top right) or radical (top middle)).^[^ ^6^ ^]^

Here we report our investigation of the coordination properties of a model triphosphanyl scaffold **1** which may act variably as **1** ^−^, **1** ^∙^, or **1** ^+^ (**Figure** **1**) towards a series of transition metal carbonyls [M(CO)_x_]^z−^ (M = Mn (x = 5, z = 1), Fe (x = 4, z = 2) or Co (x = 4, z = 1)) with gradually increasing electron number in the valence shell of the metal fragments to foster the availability for electron transfer and ultimately bond activation. For our study the above mentioned ferrocene bridged triphosphanyl scaffold **1** seems to be an ideal choice, since the number of stereoisomers caused by the presence of *P*‐stereogenic centers is limited. On top of that the anionic, cationic and electroneutral (radical) forms are known and synthetically available and the electron number varies at the central phosphorus atom (Figure 1).^[^ ^6^ ^]^

According to recent studies, exclusive coordination at the terminal phosphorus atoms is observed for the anionic P_3_ unit in **1** ^−^ (e.g., in **Li**‐**1**), whereas the neutral radical **1** ^∙^ undergoes dimerization via the central phosphorus atom and the reactivity of the cationic bisphosphanyl phosphenium ion **1** ^+^ can involve both positions.^[^ ^6^, ^7^ ^]^

## 2 Results and Discussion

### 2.1 Coordination of the P_3_‐Unit

**Scheme 1:**
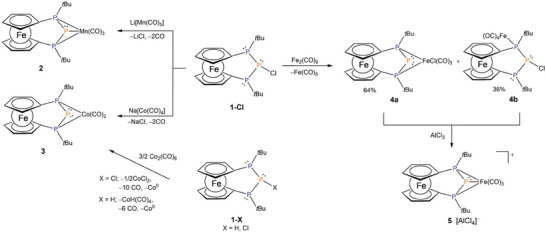
Synthetic access to the P_3_ metal complexes starting from functionalized bisphosphanylphosphanes **1‐Cl** and **1‐H**.

Anticipating formation of a phosphenium transition metal complex we reacted **1**‐**Cl** with Li[Mn(CO)_5_] as anionic metal fragment (**Scheme** **1**). A straightforward metathesis reaction with loss of two CO ligands was observed. ^31^P NMR data of the product confirm the integrity of the P_3_‐unit. The central P atom resonates at −332.5 ppm (t, ^1^ *J*_PP_ = 345 Hz) and the terminal P atoms at 54.4 ppm (d). The three carbonyl ligands in manganese complex **2** are chemically equivalent showing a single resonance at 226.9 ppm in the ^13^C NMR spectrum. The IR spectrum reveals two CO bands at 1984 and 1907 cm^−1^, indicating a high symmetry and the stereospecific formation of the *fac‐*isomer of **2**. Identity and purity of **2** were further confirmed by ^1^H‐, ^13^C‐NMR spectroscopy, mass spectrometry, elemental analysis and X‐ray crystallography.

To finetune the electronic situation we explored an analogous reaction with the more electron rich 18 valence electron fragment [Co(CO)_4_]^−^ by reacting **1**‐**Cl** with Na[Co(CO)_4_] (Scheme 1). Again, CO is eliminated during coordination, and the terminal phosphorus atoms remain chemically equivalent featuring a ^31^P‐NMR resonance at 65.1 ppm (d) which is very similar and only slightly low‐field shifted compared with Mn‐complex **2**. By contrast, the central phosphorus atom (δ(^31^P): −285.9 ppm (t, ^1^ *J*_PP_ = 377 Hz) representing the formal phosphanide center in **3** experiences deshielding by 45 ppm compared with the phosphorus atom in the analogous position in **2**. For comparison, similar values (−250.8 and 30.0 ppm) were reported by Hey‐Hawkins and Wolf in case of an anionic carborane substituted triphospholane cobalt complex.^[^ ^2^ ^]^ For the two carbonyl ligands in **3** two broad resonances are found at 219.8 and 208.5 ppm in the corresponding ^13^C‐NMR spectrum, indicating a tetragonal geometry at the Co‐metal center or the occurrence of a *rac* diastereomer of the [3]ferrocenophane ligand in the case of a quadratic planar coordination polyhedron. The presence of only two carbonyl bands at 1970 and 1913 cm^−1^ in the IR spectrum verifies the loss of the other two CO ligands. Also, for Co‐complex **3** identity and purity were corroborated by ^1^H‐, ^13^C‐NMR spectroscopy, mass spectrometry and elemental analysis. Finally, it is worth mentioning that the same cobalt complex **3** was formed by reacting **1**‐**Cl** or **1**‐**H** with 1.5 equivalents of dicobaltoctacarbonyl.

**Figure 2:**
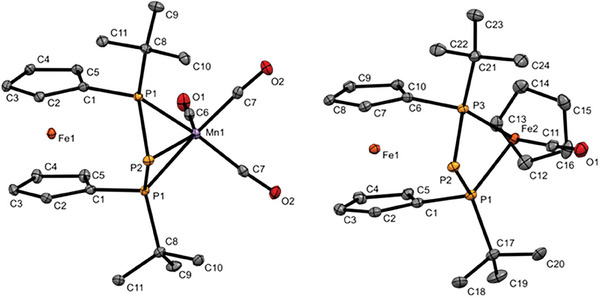
Molecular structures of **2** and **7** in the solid state. Ellipsoids are shown at 30% probability and all hydrogen atoms are omitted for clarity. Selected bond lengths and angles in **2**: 2.1463(7) Å (P1‐P2), 2.2493(6) Å (Mn1‐P1), 2.5692(8) Å (Mn1‐P2), α = 4.3(1)°, τ = 0.0°); 7: 2.1700(9) Å (P1‐P2), 2.1694(8) Å (P2‐P3), 2.2416(8) Å (Fe2‐P3), α = 4.6(1)°, τ = 0.2°).

Based on the above discussed spectroscopic data the coordination of the two terminal phosphorus atoms of the P_3_‐unit to the metal fragments is indisputable, in agreement with the fact that the 14 valence electron metal fragments [Co(CO)_2_]^−^ and [Mn(CO)_3_]^−^ are prone to additional coordination to complete their 18 valence electron shell. On the other hand, the question arises whether the central phosphorus coordinates to the metal fragments. In order to get deeper insight into the coordination mode of **2** and **3** DFT calculations were performed (at ωB97X‐D/def2‐TZVP level of theory, more details in SI). Bader analysis verified the expected η^2^‐coordination mode, since no bond critical point (BCP) was found between the central P and the metal fragments, while bond critical points with an electron density of 0.080 au for **2** and 0.088 au for **3** were localized between the terminal phosphorus and the metal centers. The strong coordination of the two outer phosphorus atoms to the metal fragments was verified by the Wiberg bond indices as well (**Table** **1**). Interestingly, in case of **2** the calculated Wiberg bond index (WBI) for the bond between the central phosphorus and the manganese center is 0.731, indicating coordination of the third, central phosphorus as well. In agreement with the weaker interaction the calculated bond length between the central phosphorus and manganese center is significantly longer (≈2.62 Å), than in case of terminal phosphorus (≈2.27 Å) (cf. **Figure** **2** and the discussion of the obtained X‐ray structures for experimental bond lengths). The Wiberg bond index is 0.224 for the analogous metal‐phosphorus bond in case of **3**, showing a much weaker interaction, in full agreement with the more electron rich character of the cobalt fragment. Natural bond orbital (NBO) analysis further bolstered the different character of the two complexes. In essence, the Wiberg bond indices suggest an η^3^‐coordination mode in case of Mn complex **2**, with a weaker bond between the central phosphorus and Mn center, while for the analogous position in Co complex **3** no bond was found. On top of that second order perturbation theory analysis of the Fock matrix on NBO basis shows only very minor interaction (1–2 kcal mol^−1^) between the phosphanide center and the cobalt atom. The analysis of the frontier orbitals shows that the HOMO is mainly localized at the central phosphorus atom, while the Co(CO)_2_ fragment makes a significant contribution to the LUMO (Figure S1, Supporting Information). With regard to the classical 18 valence electron rule both fragments, [Mn(CO)_3_] and [Co(CO)_2_], are isoelectronic and would be anticipated to behave similarly. The values of the measured and calculated vibrational frequencies are more consistent with positively charged metal atoms,^[^ ^8^ ^]^ therefore in this manuscript we refrain from allocating formal charges to the atoms, as this would result in negative charges assigned to the metal cations, in contrast to the actual findings.

**Table 1:** Survey of Selected Experimental Spectroscopic and Structural Data and Calculated Bonding Parameters of **1‐Li**, **2**, **3**, **5**, and **7**.

		1‐Li	2	3	5	7
Metal (M)		Li	Mn	Co	Fe	Fe
ρ(BCP)^[^ ^a^ ^]^ [au]	M–*P*_outer_	0.023	0.080	0.088	0.086	0.087
	M–*P*_central_	–	–	–	0.060	–
WBI	M–*P*_outer_	0.418	0.904	0.818	0.903	0.906
	M–*P*_central_	0.387	0.731	0.224	0.859	0.201
Occupancy of NBO	M–*P*_outer_	–	1.846	1.834	1.825	1.883
	M–*P*_central_	–	1.734	–	1.721	–
d(M–P) (calc.) [Å]	M–*P*_outer_	2.359	2.270	2.257	2.257	2.270
	M–*P*_central_	2.556	2.619	3.237	2.483	3.355
δ(^31^P) [ppm]	*P*_outer_	14.9	54.4	65.1	65.4	36.0 36.2
	*P*_central_	−116.4	−332.5	−285.9	−267.4	52.0
^1^ *J*_PP_ [Hz]		271	345	377	401	249 270

^[a]^ Density of all electrons at the bond critical point (ρ(BCP).

Since the two metal complexes **2** and **3** show different coordination modes we were puzzled to obtain a valence isoelectronic iron complex. To this end, **1**‐**Cl** was reacted with Collman`s reagent which unfortunately lead to unselective reactivity only. Hence, we changed our synthetic strategy and performed a two‐step reaction with initial coordination to a neutral iron carbonyl fragment and subsequent chloride abstraction (Scheme 1). The first reaction step leads to a mixture of two transition metal complexes involving either η^2^‐ (**4a** (64%) or η^1^‐ **4b** (36%)) coordination to the metal center. In addition, a transfer of the Cl‐ligand from the central phosphorus atom to the Fe(CO)_3_‐unit was observed in **4a**, which is a known phenomenon already observed in structurally related Fe(0)‐complexes before.^[^ ^7^ ^]^ In full agreement with the isolation of **4a** and **4b** the thermodynamic driving force of the formation of two compounds is similar as it was demonstrated by the thermoneutral **4b** → **4a** + CO hypothetical reaction (Δ*G* = 3.2 kcal mol^−1^). The direct transformation between **4a** and **4b** can be excluded as we were not able to find any monomolecular transition state between the two isomers. Due to similar physical properties the mixture of **4a** and **4b** could not be separated. In order to get the desired product, the mixture was reacted in situ with the halide scavenger AlCl_3_ leading to quantitative formation of cationic binuclear Fe‐complex **5**[AlCl_4_] (Scheme 1). As expected, the transformation of **4a** and **4b** is exergonic.

In complex **5**[AlCl_4_] the terminal phosphorus atoms are chemically equivalent with a chemical shift (δ(^31^P): 65.4 ppm (d)) very close to their counterparts in complexes **2** and **3**. The central phosphorus atom in cationic Fe complex **5** reveals the most downfield shifted signal (δ(^31^P): –267.4 ppm) for the formal phosphanide center in this series of metal carbonyl complexes **2**, **3** and **5**[AlCl_4_], along with the largest value of the ^1^ *J*_PP_ coupling constant (401 Hz). Similar to the neutral Mn complex **2**, the three chemically equivalent carbonyl ligands in **5**[AlCl_4_] feature one broad resonance at 206.7 ppm in the corresponding ^13^C NMR spectrum. In contrast to the Mn complex **2** three carbonyl bands at 2068, 2023 and 2011 cm^−1^ are observed in the IR spectrum of **5**[AlCl_4_] pointing to a different coordination polyhedron. Identity and purity of Fe‐metal complex **5**[AlCl_4_] were further corroborated by ^1^H‐, ^13^C‐, ^27^Al‐NMR spectroscopy, mass spectrometry and elemental analysis. The Bader analysis, Wiberg bond indices and NBO (Table 1) unanimously suggest an η^3^‐coordination mode.

**Scheme 2:**
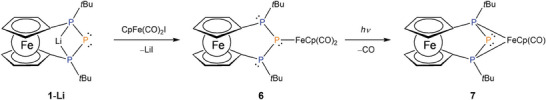
Salt metathesis reaction of lithium phosphanide **1**‐**Li** with FeICp(CO)_2_ and CO elimination under UV‐light irradiation to Fe complex **7**.

To circumvent the positive charge of the complex, the neutral Fe(II)‐complex **7** was prepared by treating lithium phosphanide **Li**‐**1** with CpFe(CO)_2_I (**Scheme** **2**). In a step‐wise reaction the mononuclear Fe complex **6** is formed as primary product, which subsequently loses one CO ligand under UV light irradiation leading to the monocarbonyl Fe(II) complex **7**. η^1^‐ η^2^−coordination modes of **6** and **7** were supported by our DFT calculations and the transformation of **6** to **7** is exergonic (Δ*G* = −22.5 kcal mol^−1^), in full agreement with the experiment, where we were not able to convert back **7** to **6** in the presence of carbon monoxide.

Contrary to the expectations the formal phosphanide center in complex **7** shows a dramatically downfield shifted ^31^P‐NMR resonance at 52.0 ppm in the spectrum for the formal phosphanide center which is more than 300 ppm deshielded compared with its counterparts in all other isolated metal carbonyl complexes reported in this work. This signal splits into a dublet of dublets owing to the direct coupling with the two chemically nonequivalent terminal phosphanyl units found at 36.0 (^1^ *J*_PP_ = 249 Hz) and 36.2 ppm (^1^ *J*_PP_ = 270 Hz) as two dublets. The resonance of the CO ligand was found as a multiplet at 220.5 ppm in the corresponding ^13^C‐NMR spectrum. Again identity and purity of the Fe(II) complex **7** were further corroborated by ^1^H‐, ^13^C‐NMR spectroscopy, IR spectroscopy, mass spectrometry, elemental analysis, and X‐ray crystallography. Contrary to cationic **5**, the P_3_ unit possesses an η^2^ coordination mode in case of **7** (Table 1). A similar initial η^1^ coordination at the central phosphorus atom transforming into η^2^ coordination to the terminal positions is in agreement with previously reported NiCp^+^ complexes of the P_3_ fragment.^[^ ^9^ ^]^

X‐ray crystallographic data confirm the structural arrangement derived from the spectroscopic data discussed before. In **2** the Mn atom adopts a distorted trigonal antiprismatic environment common for such type of complexes,^[^ ^2a^ ^]^ with *fac*‐η^3^‐coordination of the P_3_‐unit (Figure 2). The P–P bond length of 2.1463(7) Å found in **2** is slightly shorter compared with the starting material **1**‐**Cl** (2.252(5) Å (P–P)),^[^ ^10^ ^]^ but comparable to metallated triphospha [3]ferrocenophanes **1** ^−^.^[^ ^6a^ ^]^ The angular sum at the central phosphorus(III) atom (ΣP2 = 198(1)°) indicates a highly pyramidal arrangement of substituents and the electron lone pair. While the Mn‐P1 bond (to the terminal phosphorus atoms) is in the region of a Mn–P single bond (2.2493(6) Å),**^[^** ^11^ **^]^** the Mn‐P2 bond length (to the central phosphorus atom) is significantly longer (2.5692(8) Å), suggesting a rather weak chemical bond as pointed out earlier.

Similarly, X‐ray crystallographic data of **7** confirm η^2^‐coordination of the P_3_‐unit as derived from the spectroscopic data discussed before (Figure 2). The P–P bond lengths of 2.1700(9) Å (P1‐P2) and 2.1694(8) Å (P2‐P3) found in **7** are comparable to Mn‐complex **2** and other metallated triphospha [3]ferrocenophanes **1** ^−^.^[^ ^6a^ ^]^ Interestingly, the transannular distance between both terminal phosphorus atoms in **7** (2.6277(10) Å (P1∙∙∙P3)) is unusually short and significantly below the sum of the van der Waals radii of both atoms, unlike in Mn‐based **2** (2.9284(11) Å). While the Fe2‐P1 (2.2349(7) Å) and Fe2‐P3 (2.2416(8) Å) bonds (to the terminal phosphorus atoms) in **7** are in the range of a Fe–P single bond, the Fe2‐P2 distance is significantly longer (3.3502(9) Å), suggesting no chemical bond between the formal phosphanide center and the metal atom in contrast to Mn complex **2**.

### 2.2 Bond Activation in the P_3_‐Unit

**Scheme 3:**
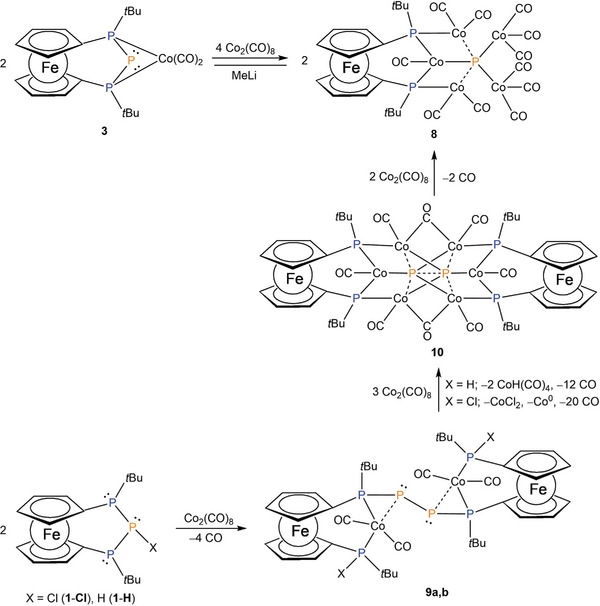
P–P bond activation in complex **3** by treatment of additional Co_2_(CO)_8_ and stepwise bond activation in triphosphane **1‐Cl** or **1‐H** after successive addition of Co_2_(CO)_8_. Considering the experimentally obtained bond lengths and the results of the DFT calculations the solid lines indicate “classical” 2c2e single bonds, while dashed lines are used in the cases, where the DFT calculations suggest interaction (presence of BCP), but significantly weaker than a 2c2e single bond. For better overview we do not show any formal or partial charges in this scheme (cf. Figure S2, Supporting Information for detailed charge values).

Co complex **3** can be converted to the Co cluster **8** by the addition of two equivalents of dicobaltoctacarbonyl (**Scheme** **3**). Here, an insertion of cobaltcarbonyl fragments into both P–P bonds takes place, whereby the formally released phosphanylidene center is trapped by five cobalt carbonyl fragments, while the remaining bisphosphanido ligand forms coordinative bonds to two different cobalt centers.

The insertion of cobalt into both P–P bonds and the formal insertion of two dicobaltheptacarbonyl fragments causes a significant downfield shift (Δδ = 532 ppm) of the signal (triplet) of the central phosphorus atom from −285.9 ppm in **3** to 246.1 ppm in **8** in the ^31^P‐NMR spectrum, while the *J*_PP_ coupling to the two chemically equivalent phosphanido centers drops from ^1^ *J*_PP_ = 377 Hz in **3** to ^2^ *J*_PP_ = 168 Hz in **8**. The signal of the two outer phosphanido centers is detected marginally high field shifted compared to the starting complex **3** as a doublet at 43.5 ppm. While the latter signal is well resolved in the ^31^P NMR spectrum, the signal from the central phosphorus atom is significantly broadened due to the quadrupolar nature of the adjacent ^59^Co nuclei.

The IR spectrum of **8** shows four distinct bands at 2070, 2027, 1989 and 1939 cm^−1^ for the CO vibrations, while some bands are overlapping and are not baseline resolved. Identity and purity of cobalt cluster **8** were further confirmed by ^1^H‐ and ^13^C‐NMR spectroscopy, mass spectrometry (MALDI), elemental analysis, and X‐ray crystallography.

**Figure 3:**
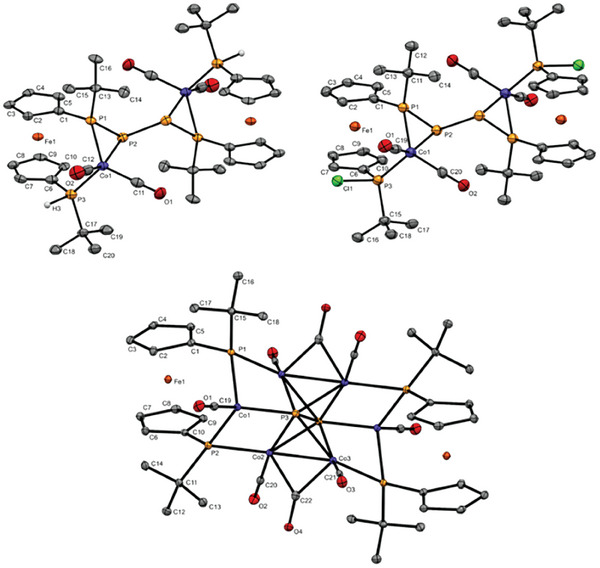
Molecular structure of **8** in the solid state (left) and an excerpt of the P_3_Co_5_ unit (right). Ellipsoids are shown at 30% probability and all hydrogen atoms are omitted for clarity.

The molecular structure illustrated in **Figure** **3** confirms P–P bond activation with insertion of the cobalt into the phosphorus phosphorus bonds of the *ansa*‐bridge. Cobalt cluster **8** crystallizes with almost plane‐parallel arrangement of the Cp ligands (α = 5.1(5)°) and staggered conformation of the unstrained ferrocene backbone (τ = 17.2°). The *tert*‐butyl substituents are pointing to the same side of the ferrocenophane ring, similar to Mn complex **2** or Fe complex **7**.

In the cluster both terminal phosphido centers adopt a highly distorted tetrahedral coordination with bond angles ranging from 72° to 128°. The Fe—Co distance to the two proximate cobalt atoms (Co1: 4.348(2) Å; Co2: 4.471(2) Å) is in the range of the sum of the van der Waals radii of the two elements,^[^ ^12^ ^]^ indicating no interaction. The P–Co bond lengths vary between 2.11 and 2.26 Å, but correspond to common literature examples for cobalt(I), cobalt(II), or cobalt(III) complexes with bridging phosphido ligands.^[^ ^4^, ^13^ ^]^ The Co–Co distances in the range of 2.58 to 2.78 Å are comparable to the Co–Co bond lengths in binuclear cobalt complexes.^[^ ^13b,d^ ^]^

For a better understanding of the bonding situation in Co‐cluster **8** Bader's analysis was carried out. Bond critical points were found between the phosphorus atoms and the metal fragments, with electron densities amounting to 0.087–0.110 au. The calculated Wiberg bond indices of these Co–P bonds are between 0.754 and 1.074, suggesting similar bond strength of these bonds in line with the experimentally obtained bond lengths. Although the Co–Co distances in **8** are similar to binuclear cobalt complexes with metal–metal bonds,^[^ ^13b^ ^]^ the lack of bond critical points and the low values of the Wiberg bond indices do not support the existence of direct Co–Co bonds in **8** (Table S7, Supporting Information).

Cluster **8** can also be derived from triphospha [3]ferrocenophanes **1**‐**Cl** or **1**‐**H**, where several intermediates could be identified (**9a, 9b, 10**) (Scheme 3), providing evidence for a stepwise conversion. The appearance of these intermediates suggests that P–P bond activation occurs in **1**‐**Cl** and **1**‐**H**, with one of the two P–P bonds oxidatively adding to the cobalt center. Simultaneously, this species dimerizes over the formerly central phosphorus atom, forming a new P–P bond (Scheme 3). It was already shown that metal‐bonded phosphanide ligands can undergo a radical P–P coupling, which leads to dimeric products.^[^ ^14^ ^]^ The third by‐product, which was obtained as single‐crystalline material, represents the dimeric cobalt complex **10**. The appearance of this species indicates further P–P bond activation, formally inserting another cobalt carbonyl fragment into one of the two P–P bonds of **9a** or **9b**, respectively.

In the case of bisphosphanyl chlorophosphane **1**‐**Cl** as starting compound, blue cobalt(II) chloride was observed as a by‐product, which was identified by SC‐XRD. It is not clear in which way the two phosphorus‐bonded protons in **9a** are eliminated, but most likely they are transferred to further Co‐carbonyl and consistently cobalt tetracarbonyl hydride would be formed as a by‐product. In addition, **10** is poorly soluble in organic solvents, as a few isolated single crystals could not be completely dissolved in 0.6 mL dichloromethane. This is one reason why no signals for **10** were found in NMR spectroscopic investigations of the reaction mixtures.

**Figure 4:**
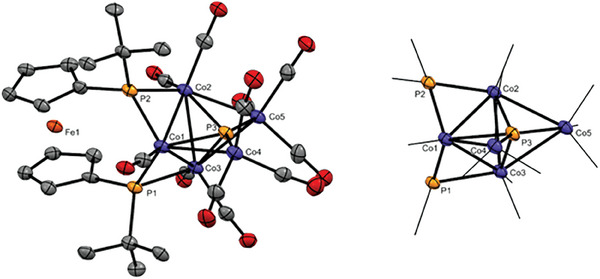
Molecular structures of Co‐complexes **9a** (top left), **9b** (top right) and **10** (bottom) in the solid state. Ellipsoids are shown at 30% probability and all except the phosphorus bonded hydrogen atoms are omitted for clarity. A disorder of the two phosphorus atoms P3 and P3', as well as the protons and *tert*‐butyl groups bound to them over two positions in **9a** is also not shown. Selected bond angles in **9a**: α = 3.7(1)°, τ = 28.6°; **9b**: α = 1.5(1)°, τ =29.2°; **10**: α = 1.0(1)°, τ = 23.0°.

Based on SC‐XRD (**Figure** **4**), the P–Co bond lengths in **9a,b** and **10** are all similar (2.1–2.2 Å) and comparable to literature known examples.^[^ ^15^ ^]^ An exception is the P_2_ fragment trapped by six Co‐metal centers in **10**, which shows a significantly elongated distance of 2.4008(8) Å to the more distant cobalt atoms P3‐Co1 as well as the P2‐Co1 bonds in **9a**,**b**, which are also significantly elongated (2.3577(2) Å (**9a**) and 2.3574(2) Å (**9b**)). The elongated P‐Co bond distances found in **9a,b** and **10** indicate rather weak interactions. While the P–P bond lengths P2‐P2' amounting to 2.2259(2) Å in **9a** and 2.2229(1) Å in **9b** correspond to classical P–P single bonds, the P1‐P2 bond distances in **9a** (2.1261(2) Å) and **9b** (2.1245(2) Å) are significantly decreased and comparable to the P–P bond length found in the phosphanide manganese complex **2** or other metalated triphospha [3]ferrocenophanes.^[^ ^6a^ ^]^ A remarkably elongated P–P bond length between both central phosphorus atoms (2.5325(15) Å (P3‐P3') is found in **10**.

Consistent with this is the analysis of the bonding situation in **9a,b** and **10** using DFT calculations (Tables S8–S10). Thus, the electron density in the bond critical points of the P1‐P2 bonds with 0.128 au and the P2‐P2' bonds with 0.114 au (for **9a**) and 0.115 au (for **9b)** is identical in **9a** and **9b**. The barely lower electron density in the bond critical point of the P2‐P2' bond indicates a slightly weaker bond, which correlates with the slightly longer P2‐P2' bond distance compared to the P1‐P2 bond length in **9a** and **9b**. The electron density of the bond critical points of the P–Co bonds in **9a**,**b** varies between 0.072 and 0.098 au, whereby it is apparent that the value found for P2‐Co1 and P2“‐Co1” is the lowest. Thus, these two bonds possess the weakest and at the same time the longest P–Co bonds in the two Co complexes **9a**,**b**.

The bonding situation is more complex in case of compound **10**. Similar to **8,** bond critical points were found between the Co fragments and the phosphorus atoms, but the electron densities in the BCPs cover a wide range (0.064–0.109) indicating large differences between the strength of these bonds. The weakest cobalt‐phosphorus bonds are the P3‐Co2 bonds, which was verified by the low Wiberg bond index (0.552). This correlates with the found, clearly elongated P3‐Co2 distance in **10** and indicates rather weak P‐Co interactions in this case. It is worth mentioning that in case of **8** the similar bonds (P3‐Co2 and P3‐Co3) are the weakest among the Co‐P bonds as well (WBI: 0.754 and 0.760, respectively). Very interestingly, a bond critical point was found between the two central phosphorus atoms, however the low electron density (0.057 au) and the 0.328 Wiberg bond index suggest a very week interaction between the two atoms. Considering these results, the bonding situation can be represented by the structure in Scheme 3, where the solid lines indicate “classical” 2c2e single bonds, while dashed lines are used in the cases, where the DFT calculations suggest interaction (presence of bond critical point), but significantly weaker than a 2c2e single bond. All attempts to isolate cobalt complexes **9a**,**b** and **10** on a preparative scale failed in our hands owing to similar solubility and tendency for crystallization. However, a small crop of single crystals from **9a**,**b** and **10** suitable for X‐ray analysis was obtained by crystallization directly from solutions of the reaction mixture.

Complex **8** contains a fragmented P_3_‐unit with one phosphorus atom only surrounded by metal atoms. We wondered whether this isolated P‐atom may be available for further functionalization and set out to explore its general reactivity. Based on the electronegativity difference of the elements involved a phosphidic nature of the isolated phosphorus atom can be anticipated, on the other hand the calculated natural charges suggest rather a positive charge of the central P atom. In full agreement **8** does not react with trimethylsilyl chloride or methyl iodide (excess and stoichiometric). On the other hand the electrophilic nature of this phosphorus atom – in line with a description as fragmented phosphenium ion complex – was verified experimentally. By inverting the reagent polarity, nucleophilic reagents such as triethylamine, fluoride‐ or hydride sources like tetrabutylammonium fluoride or lithium triethylborohydride reacted with complex **8**. However, the complex product mixtures precluded isolation and unambiguous identification of individual products. By contrast, using methyl lithium as nucleophile led to P–P bond formation with partial regeneration of the P_3_‐unit. Prolonged reaction time (72 h) at elevated temperature (50 °C) resulted even in partial back formation of the starting material **3**. Although this is accompanied by several by‐products, this reaction proofs the reversible nature of the P–P bond activation by the Co‐carbonyl fragments involved.

## 3 Conclusion

In conclusion we have demonstrated that coordination of isovalence electronic metal carbonyl fragments to a stereochemically defined P_3_‐unit embedded into a [3]ferrocenophane scaffold is highly metal dependent. While for [Mn(CO)_3_] η^3^‐coordination to all phosphorus atoms of the chain is observed, the [Co(CO)_2_] fragment adopts an η^2^‐coordination mode. For [Fe(CO)_4_] and [FeCl(CO)_3_]^−^ η^1^‐ and η^2^‐modes are observed with preferential coordination of the terminal positions of the P_3_‐chain which transforms to η^3^‐coordination upon elimination of a CO or chloride ligand. With the closely related [FeCp(CO)_2_] fragment initial η^1^‐coordination transforming into η^2^‐coordination on CO elimination is observed, again involving the terminal positions of the P_3_‐chain only. Interestingly, excess cobalt carbonyl leads to gradual and sequential P–P bond activation at the P_3_‐unit via P_2_ and P_1_ fragments. Remarkably this P–P bond activation is a reversible process and starting from the fully fragment P_1_‐unit in which the phosphorus atom is exclusively coordinated to metal atoms, the initial P_3_‐unit is regenerated with nucleophilic reagents such as methyllithium. The here reported reversible P–P bond activation complements previously published P–P bond activation processes,^[^ ^16^ ^]^ by proceeding reversibly in a stereospecific manner and avoiding precious metals. Besides implications for the activation of P–P bonds and modifications of elemental phosphorus in which related chain‐like motifs can be found, we believe that the aspect of reversibility will spark new possibilities in the formation of homoatomic P‐chains in a defined and possibly catalytic manner, materials for which attractive properties have been forecasted which are difficult if not impossible to obtain otherwise. In future investigations we will work on increasing the specificity of the back reaction and to utilize the formal P^+^ transfer synthetically to attain such goals.

## Conflict of Interest

The authors declare no conflict of interest.

## Supporting information

Supporting Information

## Data Availability

The data that support the findings of this study are available in the supplementary material of this article.
