# Supplementary material for: Gradual Coordination and Reversible P–P Bond Activation of a P3‐Unit with Transition Metal Carbonyls
Source: Adv Sci (Weinh). 2023 Dec 31;11(11):2306805. doi: 10.1002/advs.202306805 (PMC10953586; doi:10.1002/advs.202306805)
Supplement: Supplementary file 1 — Supporting Information [file ADVS-11-2306805-s001.pdf]

ADVANCED SCIENCE
Open Access

Supporting Information

for *Adv. Sci.*, DOI 10.1002/advs.202306805

Gradual Coordination and Reversible P–P Bond Activation of a P_3-Unit with Transition Metal Carbonyls

Roman Franz, Dalma Gál, Clemens Bruhn, Zsolt Kelemen and Rudolf Pietschnig**

Supporting Information
©Wiley-VCH 2021
69451 Weinheim, Germany

Gradual coordination and reversible P-P bond activation of a P_3-unit with transition metal carbonyls

Roman Franz, Dalma Gál, Clemens Bruhn, Zsolt Kelemen* and Rudolf Pietschnig*

Abstract: Coordination of a stereochemically defined P_3-chain to a series of transition metal carbonyls $[M(CO)x]z^{-}$ (M = Mn (x=5, z=1), Fe (x=4, z=2) or Co (x=4, z=1)) has been explored using a [3]ferrocenophane scaffold. A gradual transition from η^1-, η^2- to η^3-coordination is observed where in the η^2-mode the terminal positions of the phosphorus chain are bridged. With an excess of cobalt carbonyl successive P-P bond activation and gradual separation of the central phosphorus atoms from the phosphorus chain has been observed. This process is reversible and with suitable reagents such as methyl lithium, the P_3-unit is regenerated in stereospecific manner. The bonding situation and steps of the gradual P-P bond activation are investigated by DFT calculations as well as experimental methods (e.g. NMR spectroscopy, X-ray crystallography).

Table of Contents

Experimental Procedures........S3
Synthetic protocols and characterization........S3
X-ray crystallography........S5
Computational details........S7
UV-Vis spectra of the complexes **2**,**3**,**5** and **7**........S12
NMR spectra........S13
References........22
Author Contributions........22

Experimental Procedures

All reactions were carried out by means of standard Schlenk or glovebox techniques under inert gas atmosphere (argon). Solvents were dried over Na/K alloy before use and were freshly distilled under inert gas. Deuterated solvents for NMR-spectroscopy were dried and stored over molecular sieves. $[Fe(C_5H_4P\textit{t}Bu)_2PCl]$[1], $Na[Co(CO)_4]$[2] and $Li[Mn(CO)_5]$[3] were prepared according to literature procedures while other reagents were used as received without further purification. ^{1}H-, ^{13}C-, ^{27}Al-, and ^{31}P-NMR-data was recorded on Jeol JNM-ECZL500, Varian VNMRS-500 MHz or MR-400 MHz spectrometers at 25°C. Chemical shifts were referenced to residual protic impurities in the solvent (^{1}H) or the deuterio solvent (^{13}C) and reported relative to external $SiMe_4$ (^{1}H, ^{13}C), H_3PO_4 (85%) (^{31}P) or $Al(NO_3)_2$ (^{27}Al). APCI-DIP-HR or ESI-HR mass determinations were performed on a Finnigan LCQ Deca (*ThermoQuest*). MALDI mass spectra were recorded with an UltraFlex ToF/ToF (Bruker Daltonics, Bremen, D). An N_2 laser with 337 nm wavelength and 3 ns pulse duration was used. DCTB (*trans*-2-[3-(4-*tert*-Butylphenyl)-2-methyl-2-propenylidene]malononitrile) was used as matrix. Mass calibration was carried out immediately before sample measurement on sodium formate clusters, by the ESI-Tune Mix or polystyrene (Ag adduct) standard (*Agilent*). Elemental analyses were performed with a HEKAtech Euro EA CHNS elemental analyzer. Samples were prepared in a Sn cup and analyzed with added V_2O_5. Absorption spectra were recorded using a Shimadzu UV−1900 spectrometer in solution.
Crystallographic measurements were carried out on a *Stoe* IPDS2 or a *Stoe* StadiVari diffractometer with a STOE image plate detector and a Mo-Kα (λ = 0.71073 Å) monochromator or a *Stoe* StadiVari diffractometer with a Pilatus 200K image plate detector and Cu-Kα (λ = 1.54186 Å) radiation. Direct methods were used to solve the measurements and refined by "least-square" cycles (SHELXL-2017).[4] All non-hydrogen atoms were anisotropically refined without restriction. The evaluation of the data sets, as well as the graphical preparation of the structures was carried out using Olex2[5] and Mercury.[6] Details of the structure determinations and refinement are summarized in Table S1 and Table S2. Complex **2** crystallizes in the orthorhombic space group P_{nma} with half a molecule in the asymmetric unit. The entire molecular is generated by symmetry operations. The CCDC depositions 2285944-2285949 contain the supplementary crystallographic data for this paper, which can be obtained free of charge via emailing data_request@ccdc.cam.ac.uk, or by contacting The Cambridge Crystallographic Data Centre at 12 Union Road, Cambridge CB2 1EZ, UK; fax: +44 1223 336033.

Synthetic protocols and characterization

Synthesis of 2
To a solution of 43 mg (0.1 mmol) **1-Cl** in 2 mL THF 1 mL of a freshly prepared $Li[Mn(CO)_5]$ solution (0.1 M in THF) was added dropwise at room temperature. The mixture was heated to 70 °C for 2h and stirred over night at room temperature. The solvent was removed under reduced pressure and the remaining solid was extracted with 10 mL toluene. The solid was removed and washed with 10 mL toluene. The toluene extract was evaporated to dryness yielding analytically pure product. Recrystallization from toluene at -20°C afforded 87% yield (46 mg, 0.09 mmol) as orange crystals.
^{1}H-NMR (400 MHz, THF-d8): δ 4.63 (m, 4H, Cp), 4.39 (m, 4H, Cp), 1.28 (m, 18H, *t*Bu). ^{13}C-NMR (101 MHz, THF-d8): δ 226.9 (br, CO), 79.8 (m, Cp), 77.1 (m, Cp), 77.0 (m, Cp C_{ipso}), 72.7 (pst, Cp), 72.5 (pst, Cp), 35.6 (m, *t*Bu C_q), 29.9 (m, *t*Bu). ^{31}P-NMR (202 MHz, THF-d8): δ 54.4 (d, $^{1}J_{PP}$=345 Hz), –332.5 (t, $^{1}J_{PP}$=345 Hz). (AT-IR) [cm^{-1}]: 1985 (m, CO), 1907 (s, CO).
MS (APCI-DIP-HR) [*m/z*]: 530.989770 ($[M+H]^+$ 10%), calculated for $[C_{21}H_{27}FeMnO_3P_3]^+$ = 530.989747.
Elemental analysis [%]: calculated: C, 47.58; H, 4.94, found: C 47.62, H 5.10.

Synthesis of 3
To a suspension of 43 mg (0.1 mmol) **1-Cl** and 22 mg $Na[Co(CO)_4]$ (0.11 mmol) in 10 mL *n*-pentane 1 mL of THF was added dropwise at room temperature and the reaction mixture was stirred for 4 h. The solvent was removed under reduced pressure and the remaining viscous oil was extracted with 20 mL *n*-pentane. The solid was removed

and discard. The *n*-pentane extract was evaporated to dryness yielding analytically pure product in 40% yield (20 mg, 0.04 mmol) as a deep red oil.
^{1}H-NMR (400 MHz, C_6D_6): δ 4.53 (m, 2H, Cp), 4.19 (m, 2H, Cp), 3.82 (m, 2H, Cp), 3.80 (m, 2H, Cp), 1.17 (m, 18H, *t*Bu). ^{13}C-NMR (101 MHz, C_6D_6): δ 219.8 (br, CO), 208.5 (br, CO), 79.5 (m, Cp), 78.6 (m, Cp C_{ipso}), 77.6 (m, Cp), 71.5 (m, Cp), 71.2 (m, Cp), 34.0 (m, *t*Bu C_q), 29.7 (m, *t*Bu). ^{31}P-NMR (202 MHz, C_6D_6): δ 65.1 (d, $^{1}J_{PP}$=377 Hz), –285.9 (t, $^{1}J_{PP}$=377 Hz). (AT-IR) [cm^{-1}]: 1969 (s, CO), 1913 (s, CO). MS (APCI-DIP-HR) [*m/z*]: 506.990003 ($[M+H]^+$ 45%), calculated for $[C_{20}H_{27}CoFeO_2P_3]^+$ = 506.989983. Elemental analysis [%]: calculated: C 47.46, H 5.18, found: C 47.77, H 5.34.

Synthesis of 5[$AlCl_4$]

86 mg (0.2 mmol) **1-Cl** and 72 mg (0.2 mmol) $Fe_2(CO)_9$ were suspended in 5 mL DCM and heated to 60 °C for 3 h in a pressure Schlenk-tube. All volatile components were removed under reduced pressure. The residue was dissolved in 1 mL DCM and the solution was added to 26 mg (0.2 mmol) $AlCl_3$ at room temperature. All volatile components were removed under reduced pressure again yielding analytically pure product. (90% yield (127 mg, 0.18 mmol) as orange oil.
^{1}H-NMR (400 MHz, DCM-d2): δ 4.92 (m, 2H, Cp), 4.75 (m, 2H, Cp), 4.64 (m, 4H, Cp), 1.40 (m, 18H, *t*Bu). ^{13}C-NMR (101 MHz, DCM-d2): δ 206.7 (br, CO), 79.4 (m, Cp), 78.8 (m, Cp), 75.2 (m, Cp), 74.8 (m, Cp), 68.5 (m, Cp C_{ipso}), 38.5 (m, *t*Bu C_q), 29.2 (m, *t*Bu). ^{31}P-NMR (202 MHz, DCM-d2): δ 65.4 (d, $^{1}J_{PP}$=401 Hz, $P_{terminal}$), -267.4 (t, $^{1}J_{PP}$=401 Hz, $P_{zentral}$). (AT-IR) [cm^{-1}]: 2068 (s, CO), 2023 (m, CO), 2011 (m, CO). MS (MALDI-HR) [*m/z*]: 530.9810 ($[M–(AlCl_4)]^+$ 58%), calculated for $[C_{21}H_{26}Fe_2O_3P_3]^+$ = 530.9788. Elemental analysis [%]: calculated: C 36.04, H 3.74, found: C 35.79, H 3.90.

Synthesis of 7

To a suspension of 40 mg (0.1 mmol) **1-Li** and 30 mg (0.1 mmol) FeICp(CO) in 3 mL *n*-pentane 0.5 mL THF were added at room temperature. The reaction mixture was irradiated for 5 h under UV light (150 Watt Hg-lamp). The product was formed as a solid which was separated and washed with 5 mL toluene. Evaporation of residual solvent results in analytically pure product as crystalline orange material in 41% yield (22 mg, 0.04 mmol).
^{1}H-NMR (400 MHz, DCM-d2): δ 4.99 (m, 2H, Cp), 4.90 (s, 5H, Cp), 4.52 (m, 2H, Cp), 4.36 (m, 2H, Cp), 4.32 (m, 2H, Cp), 1.23 (m, 18H, *t*Bu). ^{13}C-NMR (101 MHz, DCM-d2): δ 220.5 (m, CO), 89.9 (m, Cp C_{ipso}), 81.9 (s, Cp C_{ipso}), 80.8 (m, Cp), 78.4 (s, Cp), 71.1 (m, Cp), 70.4 (m, Cp), 36.7 (m, *t*Bu C_q), 28.7 (m, *t*Bu). ^{31}P-NMR (202 MHz, THF-d8): δ 52.0 (dd, $^{1}J_{PP}$=270 Hz, $^{1}J_{PP}$=249 Hz, $P_{central}$), 36.0 (d, $^{1}J_{PP}$=249 Hz, $P_{terminal}$), 36.2 (d, $^{1}J_{PP}$=270 Hz, $P_{terminal}$). (AT-IR) [cm^{-1}]: 1930 (s, CO). MS (APCI) [*m/z*]: 556.93 ($[M+O+H]^+$ 100%), calculated for $[C_{24}H_{32}Fe_2O_2P_3]^+$ = 557.03. Elemental analysis [%]: calculated: C 53.37, H 5.79, found: C 53.41, H 5.66.

Synthesis of 8

To a solution of 101 mg (0.2 mmol) **3** in 1 mL DCM a solution of 137 mg (0.4 mmol) $Co_2(CO)_8$ in 1 mL DCM was added dropwise at room temperature. The reaction mixture was stirred for 10 min at room temperature and all volatile components were removed under reduced pressure yielding analytically pure product. Recrystallization from a concentrated THF-solution at -20 °C afforded 35% yield (97 mg, 0.07 mmol) as black crystals.
^{1}H-NMR (400 MHz, DCM-d2): δ 5.08 (br, 6H, Cp), 4.53 (br, 4H, Cp), 4.43 (br, 6H, Cp), 0.98 (br, 36H, *t*Bu). ^{13}C-NMR (101 MHz, DCM-d2): δ 209.5 (br, CO), 206.6 (br, CO), 200.9 (br, CO), 79.1 (br, Cp), 79.1 (br, Cp), 75.1 (br, Cp), 73.5 (br, Cp), 72.9 (pst, Cp C_{ipso}), 41.7 (br, *t*Bu C_q), 27.6 (br, *t*Bu). ^{31}P-NMR (202 MHz, DCM-d2): δ 246.1 (t (br), $^{2}J_{PP}$=168 Hz, $P_{zentral}$), 43.5 (d, $^{2}J_{PP}$=168 Hz, $P_{terminal}$). (AT-IR) [cm^{-1}]: 2069 (m, CO), 2013 (s, CO), 1987 (s, CO), 1935 (s, CO). MS (MALDI) [*m/z*]: 994.578 ($[M+H]^+$ 23%), calculated for $[C_{29}H_{27}Co_5FeO_{11}P_3]^+$ = 994.677. Elemental analysis [%]: calculated: C 35.04, H 2.64, found: C 35.27, H 2.86.

X-ray crystallography

Table S1: Summary of structure determinations and refinement for **2**, **7** and **8**.

	**2**	**7**	**8**
CCDC code	2285944	2285945	2285946
Empirical formula	$C_{21}H_{26}FeMnO_3P_3$	C24H31Fe2OP3	$C_{29}H_{26}Co_5FeO_{11}P_3$, C_4H_8O
Formula weight [g/mol]	530.12	540.10	1066.01
Crystal system	orthorhombic	triclinic	monoclinic
Space group	*Pnma*	$P\bar{1}$	$P2_1/n$
Unit cell dimensions:			
a [Å]	12.0291(5)	7.6347(9)	10.4639(7)
b [Å]	17.7433(6)	9.6964(14)	15.6046(7)
c [Å]	10.5223(5)	16.864(3)	23.8373(18)
α [°]	90	76.561(12)	90
β [°]	90	78.407(12)	101.396(5)
γ [°]	90	80.895(11)	90
Volume [Å^3]	2245.84(16)	1181.3(3)	3815.5(4)
Z	4	2	4
Calculated density [g/cm^3]	1.568	1.518	1.856
Absorption coefficient μ [mm^{-1}]	1.442	1.445	2.680
F(000)	1088	560	2136
Crystal size [mm]	0.26 × 0.18 × 0.13	0.15 × 0.13 × 0.11	0.22 × 0.14 × 0.01
Radiation and λ [Å]	Mo K_α (λ = 0.71073)	Mo K_α (λ = 0.71073)	Mo Kα (λ=0.71073)
Θ-Range for data collection [°]	4.50 – 51.64	4.55 – 65.24	3.14 – 51.69
Index ranges	$-14 \leq h \leq 14$	$-11 \leq h \leq 8$	$-12 \leq h \leq 12$
	$-21 \leq k \leq 21$	$-14 \leq k \leq 14$	$-18 \leq k \leq 19$
	$-12 \leq l \leq 12$	$-25 \leq l \leq 23$	$-28 \leq l \leq 26$
Refl. collected/unique	15910/2215	14102/7228	16539/7213
Data/restraints/parameters	2215/0/142	7228/0/277	7213/0/494
Goodness-of-fit on F^2	1.082	0.991	1.051
Final R indices [I>2δ(I)]/ [wR_2]	0.0271/ 0.0675	0.0433/ 0.1053	0.0848/ 0.2362
R indices (all data)/ [wR_2]	0.0303/ 0.0689	0.0640/ 0.1131	0.1161/ 0.2610
Largest difference peak/hole [e Å^{-3}]	0.40/-0.42	0.97/-0.55	1.21/-1.15

Table S2: Summary of structure determinations and refinement for **9a**, **9b** and **10**.

	**9a**	**9b**	**10**
CCDC code	2285947	2285948	2285949
Empirical formula	$C_{40}H_{54}Co_2Fe_2O_4P_6$	$C_{40}H_{52}Cl_2Co_2Fe_2O_4P_6$	$C_{46}H_{56}Cl_4Co_6Fe_2O_8P_6$
Formula weight [g/mol]	1014.21	1083.09	1529.80
Crystal system	triclinic	monoclinic	triclinic
Space group	$P\,\bar{1}$	$C2/c$	$P\,\bar{1}$
Unit cell dimensions:			
a [Å]	10.5818(9)	18.2725(15)	10.5347(8)
b [Å]	10.7156(8)	10.5751(5)	11.2430(9)
c [Å]	12.1157(11)	24.805(2)	13.6160(11)
α [°]	70.167(7)	90	65.675(6)
β [°]	67.855(7)	113.245(6)	69.577(6)
γ [°]	60.330(6)	90	74.844(6)
Volume [Å^3]	1085.55(18)	4404.1(6)	1364.0(2)
Z	1	4	1
Calculated density [g/cm^3]	1.551	1.633	1.862
Absorption coefficient μ [mm^{-1}]	1.666	1.765	2.715
F(000)	522	2216	768
Crystal size [mm]	0.14 × 0.10 × 0.04	0.14 × 0.09 × 0.02	0.14 × 0.10 × 0.06
Radiation and λ [Å]	Mo K_α (λ = 0.71073)	Mo K_α (λ = 0.71073)	Mo K_α (λ = 0.71073)
Θ-Range for data collection [°]	3.70 – 51.22	3.57 – 51.68	3.41 – 51.39
Index ranges	$-12 \le h \le 12$	$-22 \le h \le 20$	$-12 \le h \le 12$
	$-13 \le k \le 12$	$-12 \le k \le 11$	$-13 \le k \le 13$
	$-14 \le l \le 14$	$-30 \le l \le 30$	$-16 \le l \le 16$
Refl. collected/unique	7545/4051	8441/4041	9673/5058
Data/restraints/parameters	4051/0/253	4041/0/259	5058/0/331
Goodness-of-fit on F^2	1.122	1.130	1.061
Final R indices [I>2δ(I)]/ [wR_2]	0.0776/ 0.2107	0.1028/ 0.2605	0.0281/ 0.0753
R indices (all data)/ [wR_2]	0.0955/ 0.2452	0.1389/ 0.2882	0.0362/ 0.0815
Largest difference peak/hole [e Å^{-3}]	3.47/-1.28	1.28/-0.93	0.75/-0.49

Computational details

All DFT calculations were carried out with the Gaussian 16 suite of programs.[7] In our previous studies, we showed that ωB97X-D functional describes properly similar systems.[8] Harmonic vibrational analysis was obtained at the same level, and in the case of minima, all eigenvalues of the Hessian matrix were positive. For the determination of bond critical points and for Wiberg indices, Multiwfn program was used.[9] For visualization of the molecular orbitals IQmol program was used.[10] For NBO calculations NBO 3.1 was used (implemented in Gaussian 16).

Table S3: Electron density in the bond critical point [au] and bonding index according to Wiberg in Mn complex **2** (ωB97X-D/def2-TZVP).

Bond	Electron density in bcp [au]	Wiberg bond index
P1 – P2	0.126	1.228
P2 – P3	0.126	1.228
P1 – Mn	0.080	0.904
P2 – Mn	---	0.731
P3 – Mn	0.080	0.904

Table S4: Electron density in the bond critical point [au] and bonding index according to Wiberg in Co complex **3** (ωB97X-D/def2-TZVP).

Bond	Electron density in bcp [au]	Wiberg bond index
P1 – P2	0.126	1.326
P2 – P3	0.126	1.326
P1 – Co	0.088	0.818
P2 – Co	---	0.224
P3 – Co	0.088	0.818

Table S5: Electron density in the bond critical point [au] and bonding index according to Wiberg in Fe complex **5** (ωB97X-D/def2-TZVP).

Bond	Electron density in bcp [au]	Wiberg bond index
P1 – P2	0.128	1.207
P2 – P3	0.128	1.207
P1 – Fe	0.086	0.903
P2 – Fe	0.060	0.859
P3 – Fe	0.086	0.903

Table S6: Electron density in the bond critical point [au] and bonding index according to Wiberg in Fe complex **7** (ωB97X-D/def2-TZVP).

Bond	Electron density in bcp [au]	Wiberg bond index
P1 – P2	0.124	1.281
P2 – P3	0.124	1.281
P1 – Fe	0.087	0.906
P2 – Fe	---	0.201
P3 – Fe	0.087	0.906

Table S7: Electron density in the bond critical point [au] and bonding index according to Wiberg in Co complex **8** (ωB97X-D/def2-TZVP).

Bond	Electron density in bcp [au]	Wiberg bond index
P1 – Co1	0.110	1.074
P1 – Co3	0.095	0.891
P2 – Co1	0.106	1.033
P2 – Co2	0.092	0.867
P3 – Co1	0.093	0.806
P3 – Co2	0.087	0.754
P3 – Co3	0.087	0.760
P3 – Co4	0.104	0.944
P3 – Co5	0.106	1.014
Co1 – Co2	---	0.445
Co1 – Co3	---	0.419
Co1 – Co4	---	0.100
Co2 – Co3	---	0.498
Co2 – Co5	---	0.090
Co3 – Co5	---	0.081

Table S8: Electron density in the bond critical point [au] and bonding index according to Wiberg in Co complex **9a** (ωB97X-D/def2-TZVP).

Bond	Electron density in bcp [au]	Wiberg bond index
P1 – P2	0.128	1.221
P1 – Co1	0.098	1.030
P2 – Co1	0.072	0.955
P3 – Co1	0.086	0.920
P2 – P2’	0.114	1.236

Table S9: Electron density in the bond critical point [au] and bonding index according to Wiberg in Co complex **9b** (ωB97X-D/def2-TZVP).

Bond	Electron density in bcp [au]	Wiberg bond index
P1 – P2	0.128	1.217
P1 – Co1	0.098	1.024
P2 – Co1	0.072	0.956
P3 – Co1	0.088	0.938
P2 – P2’	0.115	1.237

Table S10: Electron density in the bond critical point [au] and bonding index according to Wiberg in Co complex **10** (ωB97X-D/def2-TZVP).

Bond	Electron density in bcp [au]	Wiberg bond index
P3 – P3'	0.057	0.328
P1 – Co1	0.106	1.049
P1 – Co3'	0.092	0.863
P1' – Co1'	0.106	1.049
P1' – Co3	0.092	0.863
P2 – Co1	0.107	1.031
P2 – Co2	0.094	0.899
P2' – Co1'	0.107	1.031
P2' – Co2'	0.094	0.899
P3 – Co1	0.109	1.121
P3 – Co2	0.064	0.552
P3 – Co2'	0.087	0.805
P3 – Co3	0.086	0.797
P3 – Co3'	0.072	0.609
P3' – Co1'	0.109	1.121
P3' – Co2'	0.064	0.552
P3' – Co2	0.087	0.805
P3' – Co3'	0.086	0.797
P3' – Co3	0.072	0.609
Co1 – Co2	---	0.423
Co1 – Co3'	---	0.464
Co1' – Co2'	---	0.423
Co1' – Co3	---	0.464
Co2 – Co3	---	0.399
Co2 – Co3'	---	0.289
Co2' – Co3	---	0.289
Co2' – Co3'	---	0.399

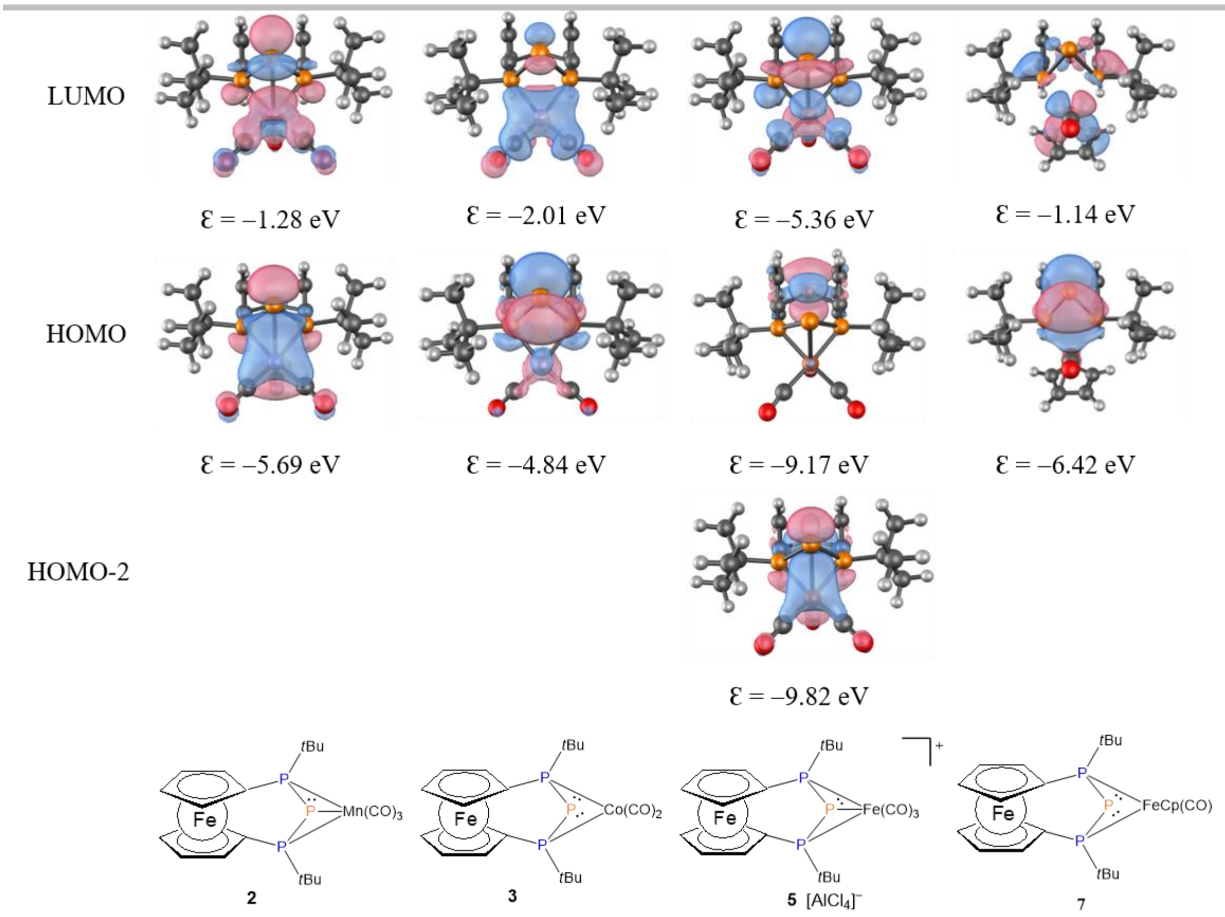

Figure S1: Kohn-Sham frontier molecular orbitals of **2**, **3, 5, 7** (ωB97X-D/def2-TZVP)

Table S11: Calculated charges of the fragments of **2** and **3** at ωB97X-D/def2-TZVP level of theory

**Total charges of the M(CO)$_n$ fragment**		
	**2**	**3**
Mulliken charge	-0.48	-0.23
APT charge	-0.54	-0.22
Natural charge	-1.12	-0.37
**Total charges of the metal centers**		
	**2**	**3**
Mulliken charge	0.10	0.16
APT charge	-1.73	-0.97
Natural charge	-2.50	-0.99

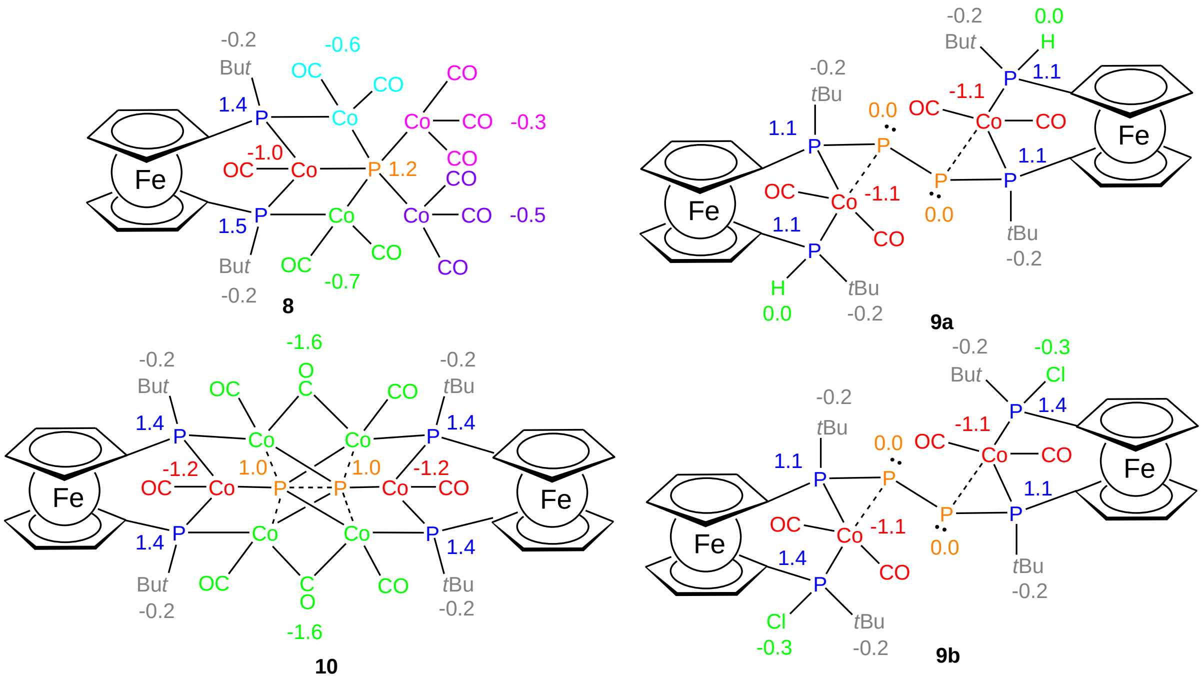

Figure S2: Calculated natural charges of the different fragments of **8**, **9a**, **9b** and **10**

UV-Vis spectra of the complexes 2,3,5 and 7:

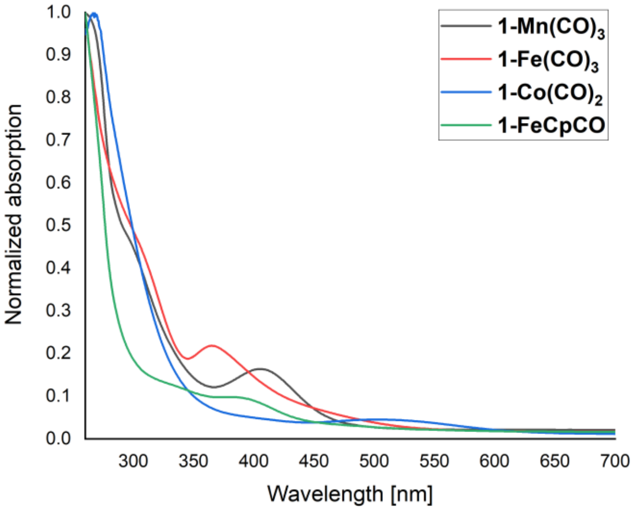

Figure S3: Absorption spectra of **2** (1-Mn$(CO)_3$ in THF), **3** (1-Co$(CO)_2$ in THF), **5** (1-Fe$(CO)_3$ in DCM) and **7** (1-FeCpCO in THF) in solution ($5 \cdot 10^{-4}$ M).

NMR spectra

On the following pages the NMR-spectra of compounds **2**, **3**, **4**, **5**[$AlCl_4$], **6**, **7** and **8** are depicted:
NMR-spectra of **2**:

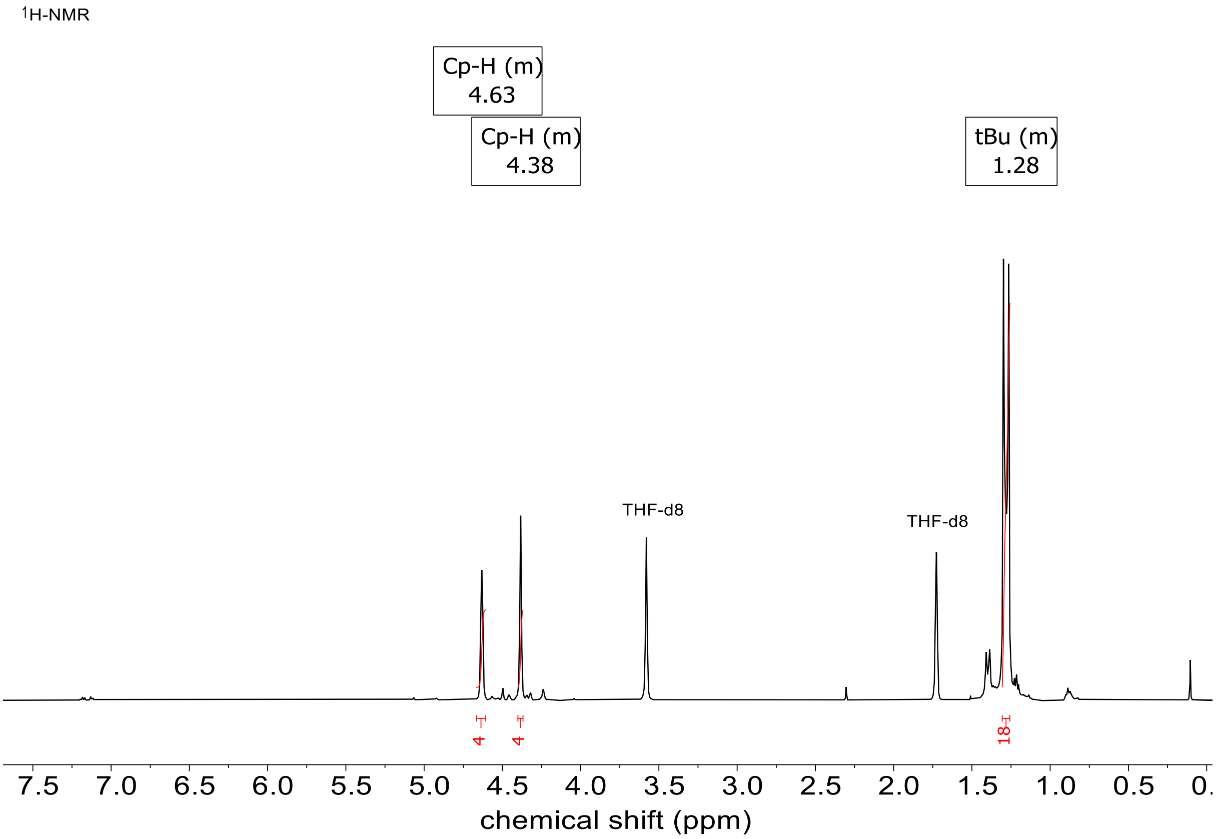

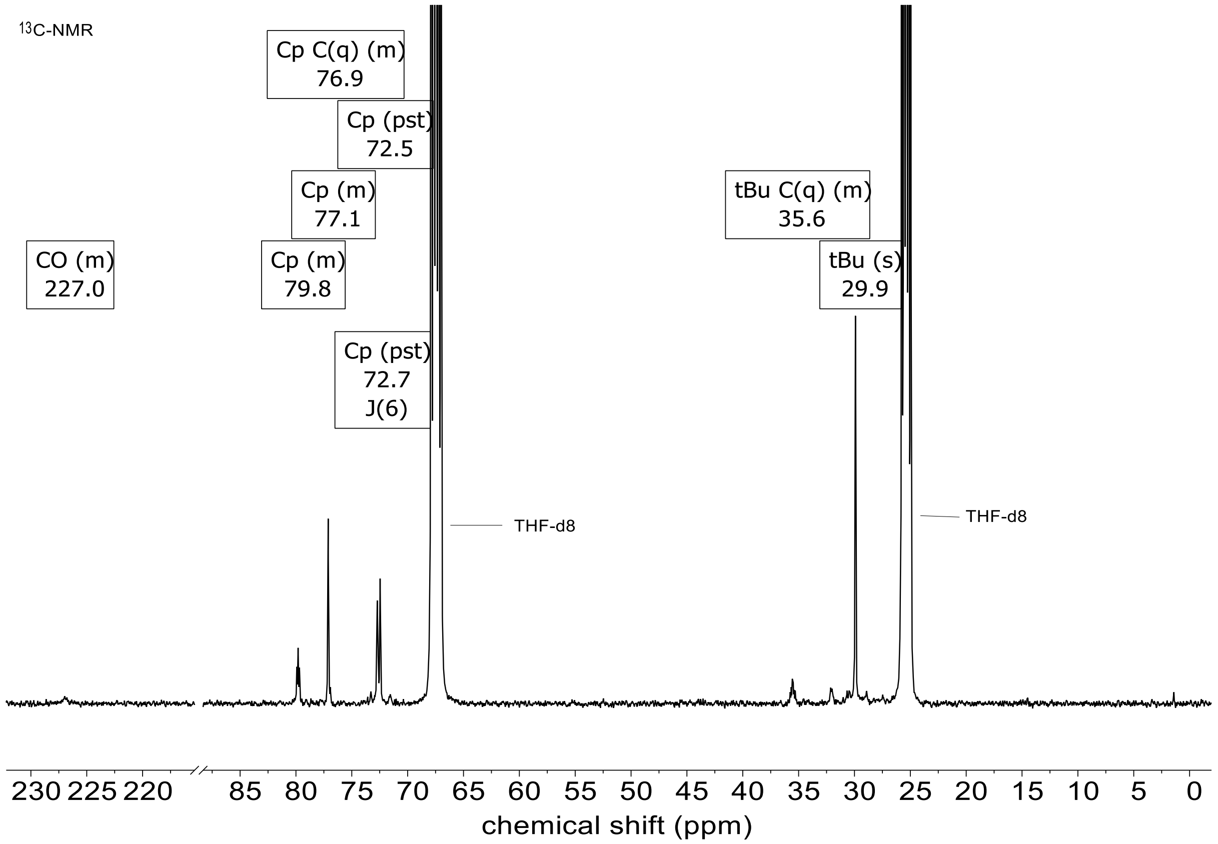

$^{31}P\{^1H\}$-NMR

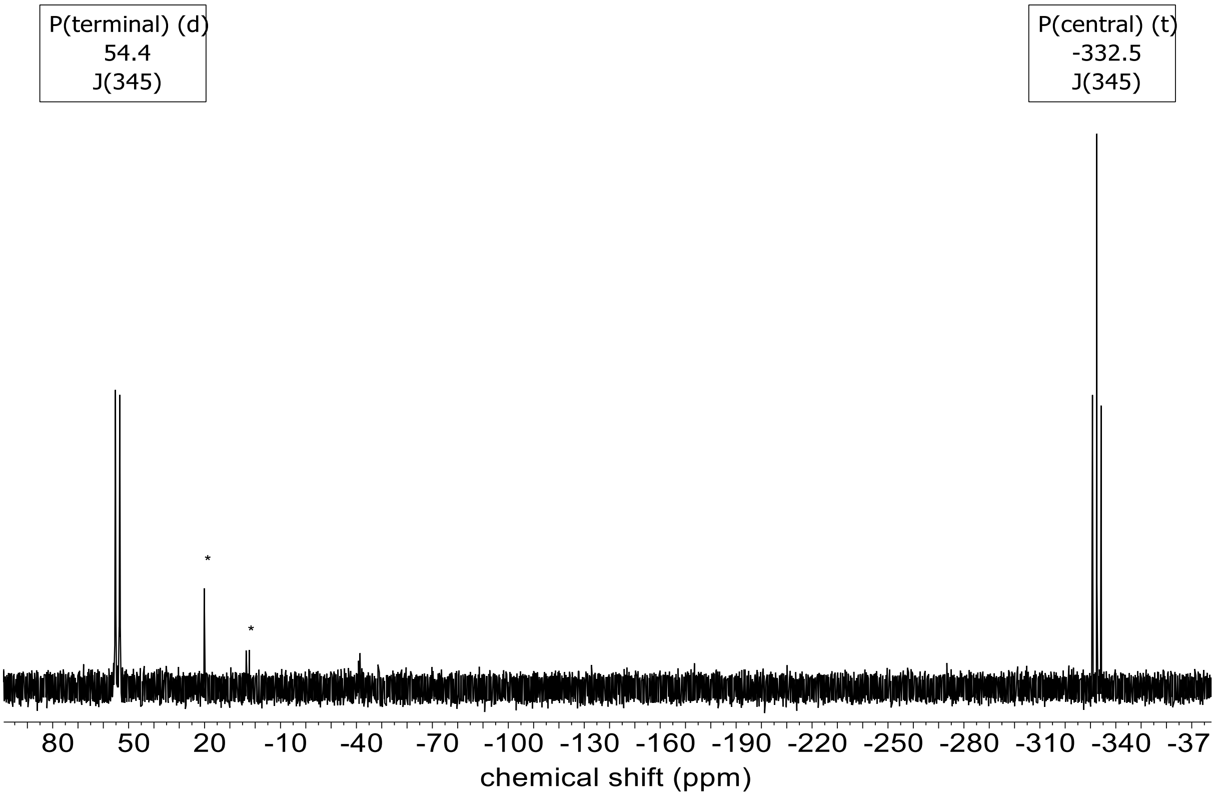

NMR-spectra of **3**:

^1H-NMR

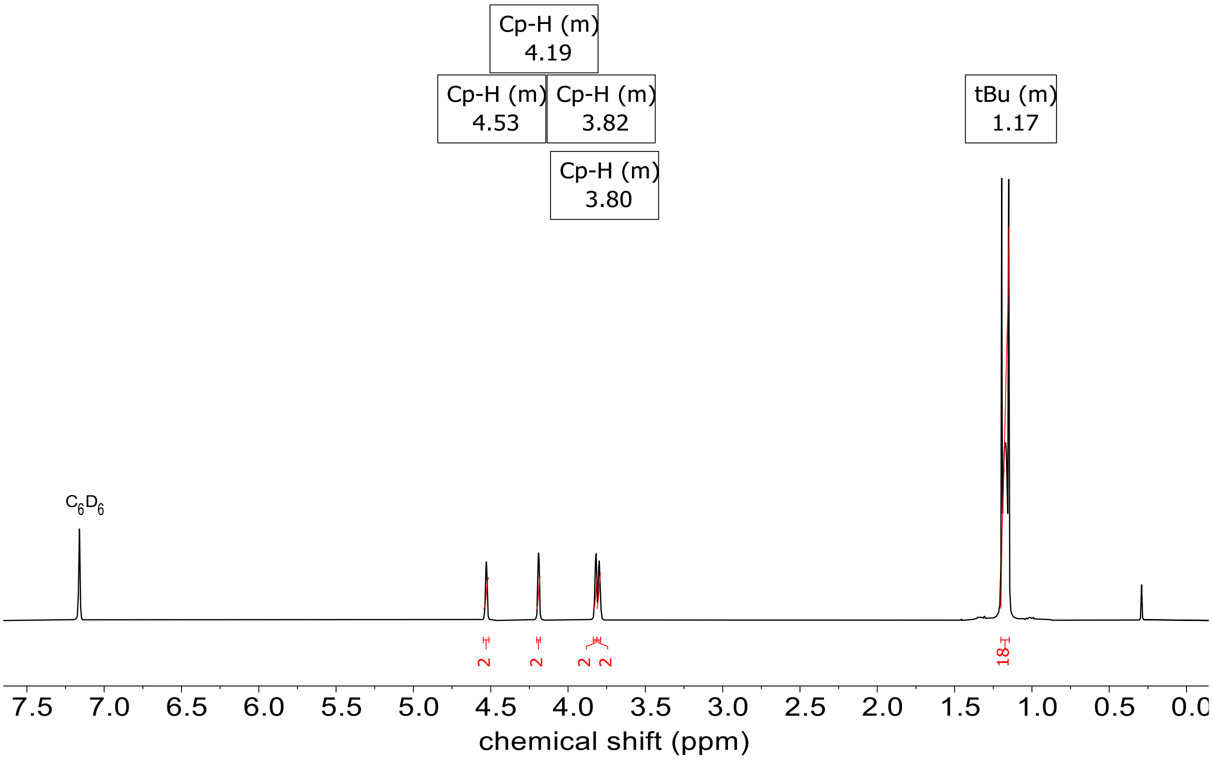

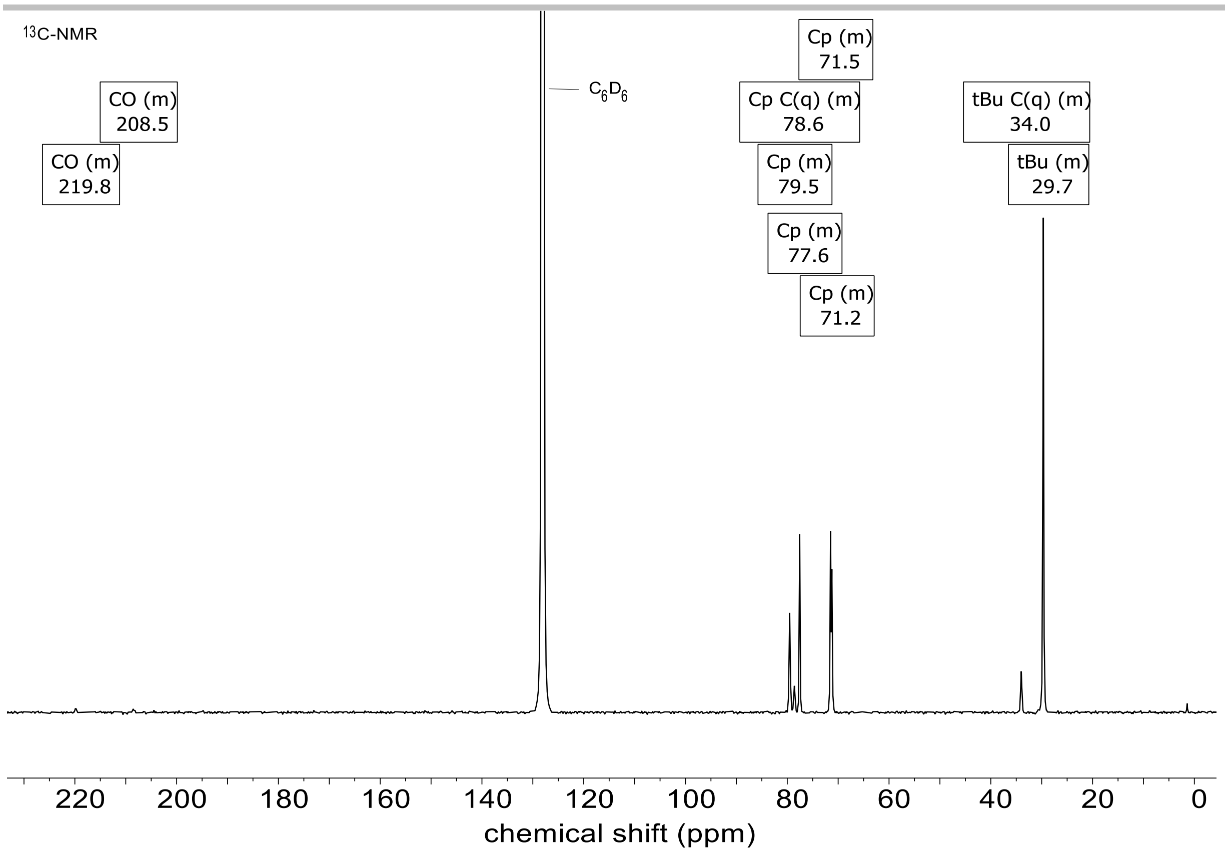
13C-NMR
CO (m) 219.8
CO (m) 208.5
C6D6
Cp (m) 71.5
Cp C(q) (m) 78.6
Cp (m) 79.5
Cp (m) 77.6
Cp (m) 71.2
tBu C(q) (m) 34.0
tBu (m) 29.7
220 200 180 160 140 120 100 80 60 40 20 0
chemical shift (ppm)

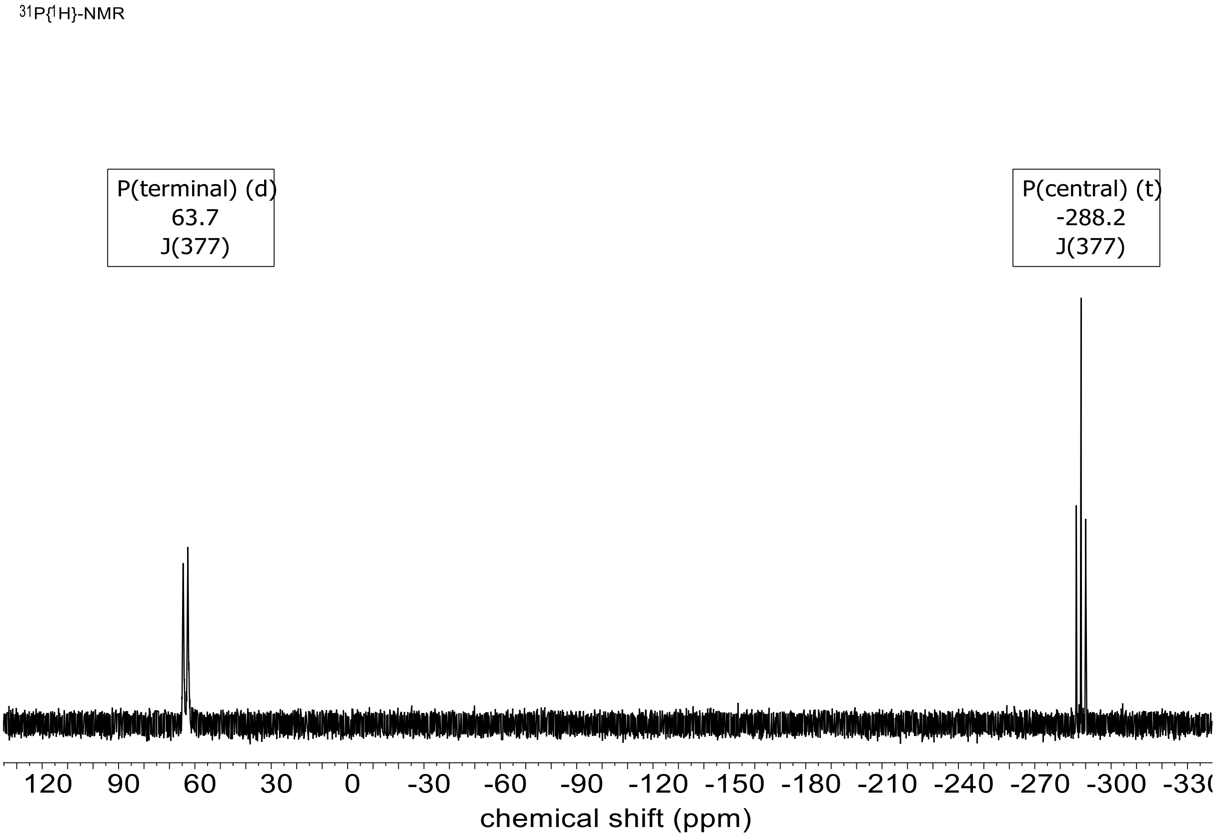
31P{1H}-NMR
P(terminal) (d) 63.7 J(377)
P(central) (t) -288.2 J(377)
120 90 60 30 0 -30 -60 -90 -120 -150 -180 -210 -240 -270 -300 -330
chemical shift (ppm)

NMR-spectra of the reaction of **1-Cl** with 1 eq. $Fe_2(CO)_9$:

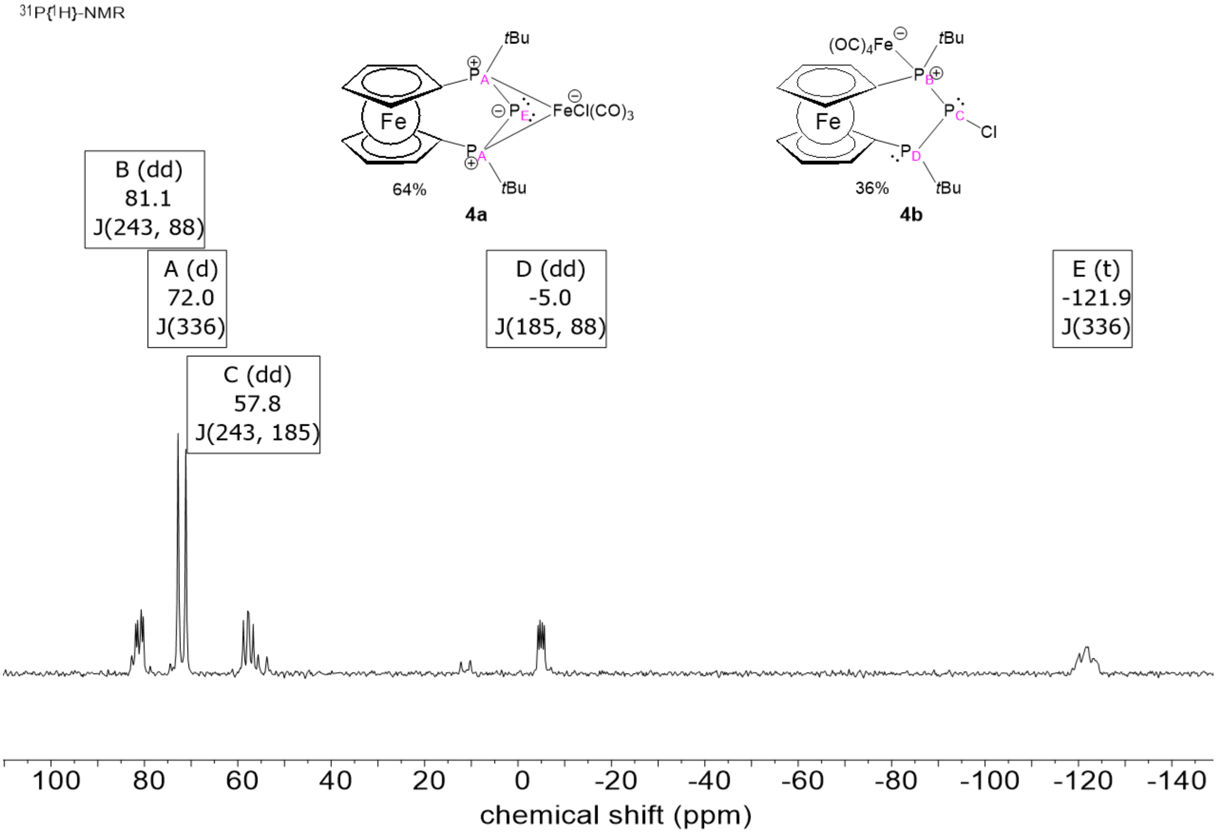

NMR-spectra of **5**[$AlCl_4$]:

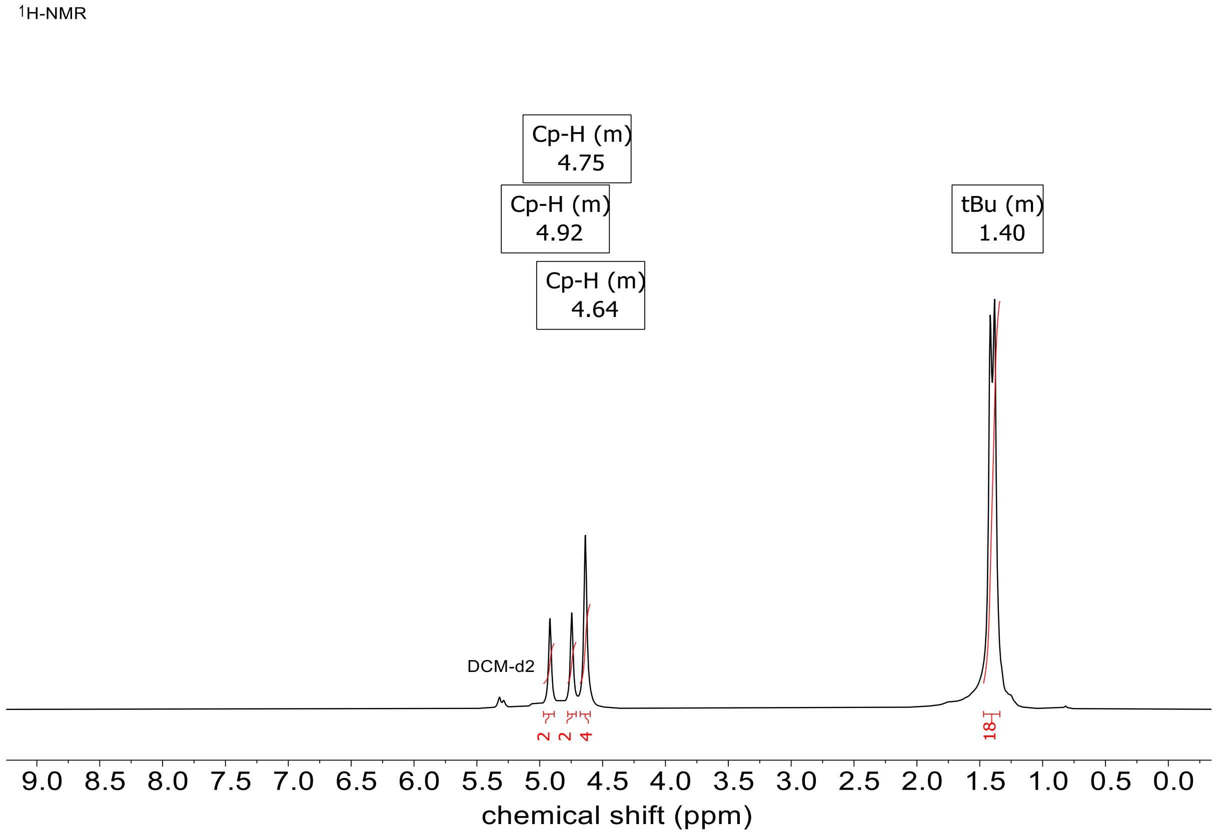

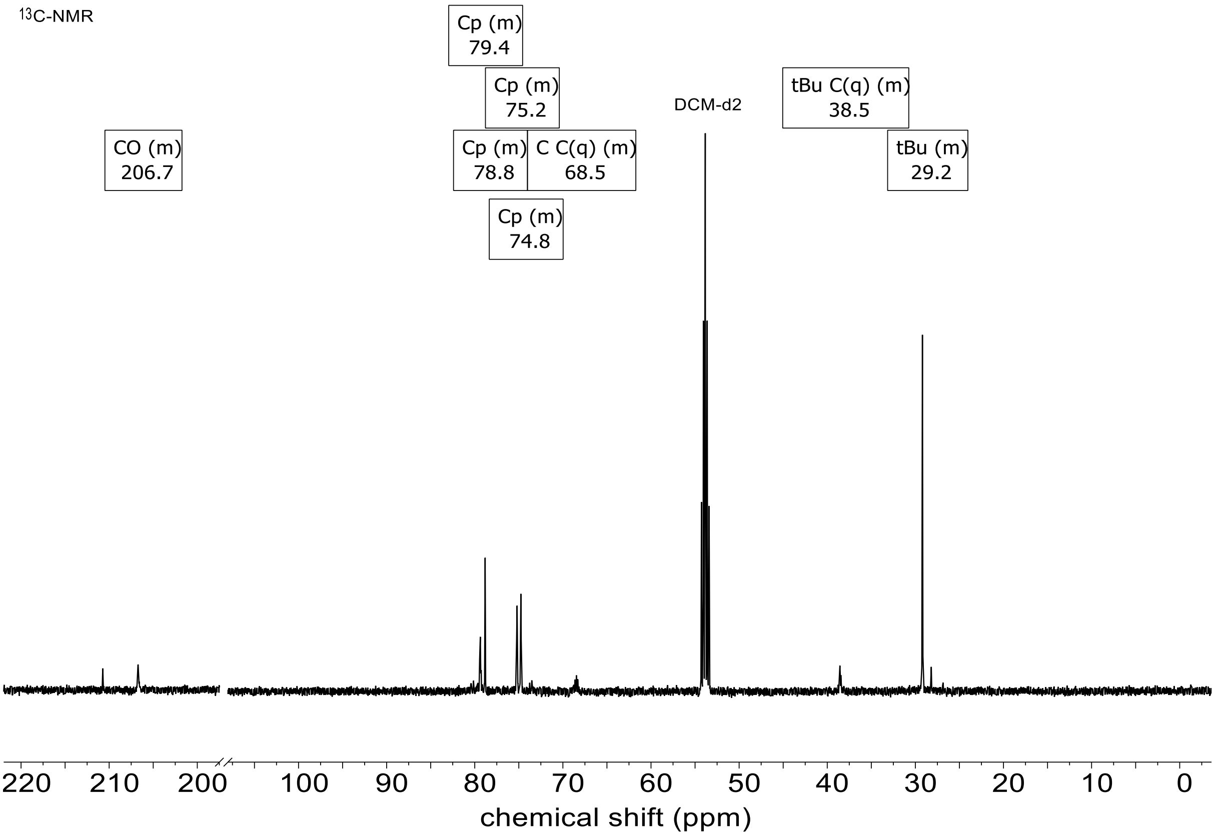
13C-NMR
Cp (m) 79.4
Cp (m) 75.2
DCM-d2
tBu C(q) (m) 38.5
CO (m) 206.7
Cp (m) 78.8
C C(q) (m) 68.5
tBu (m) 29.2
Cp (m) 74.8
220 210 200 100 90 80 70 60 50 40 30 20 10 0
chemical shift (ppm)

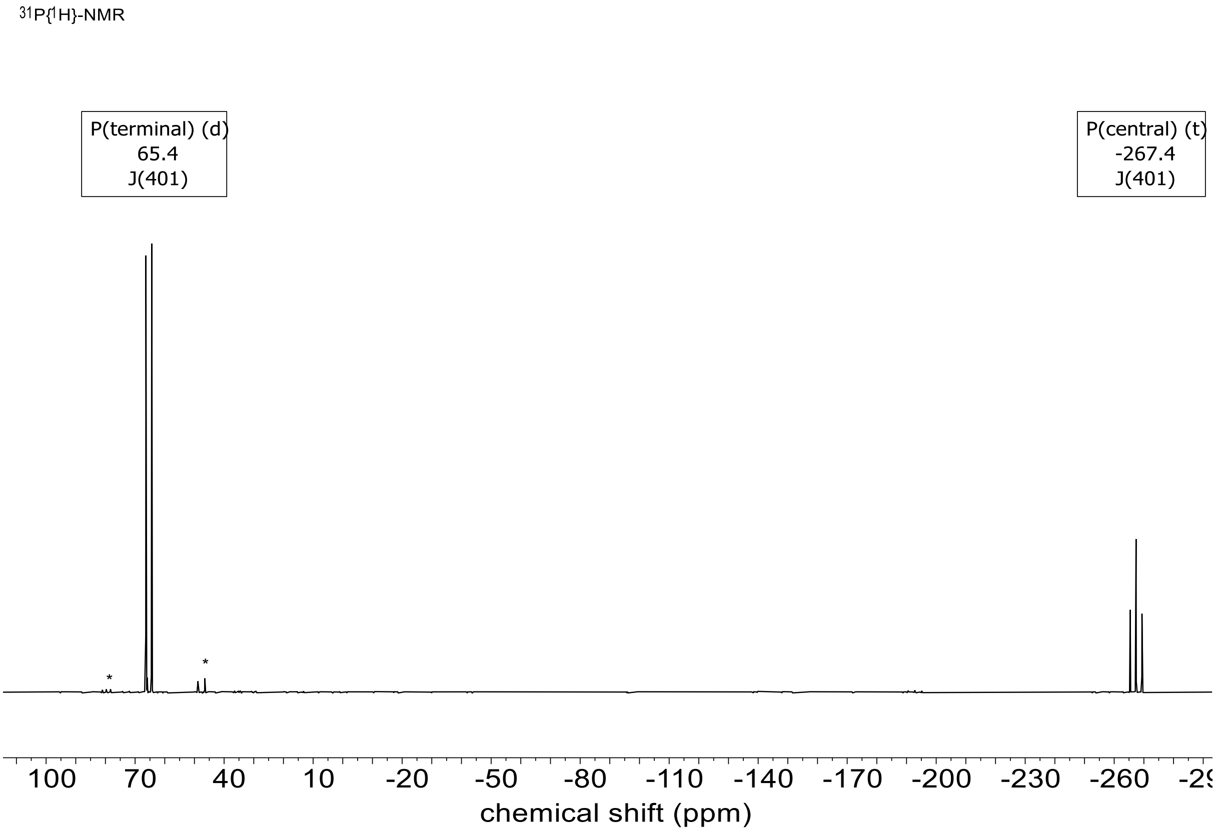
31P{1H}-NMR
P(terminal) (d) 65.4 J(401)
P(central) (t) -267.4 J(401)
*
*
100 70 40 10 -20 -50 -80 -110 -140 -170 -200 -230 -260
chemical shift (ppm)

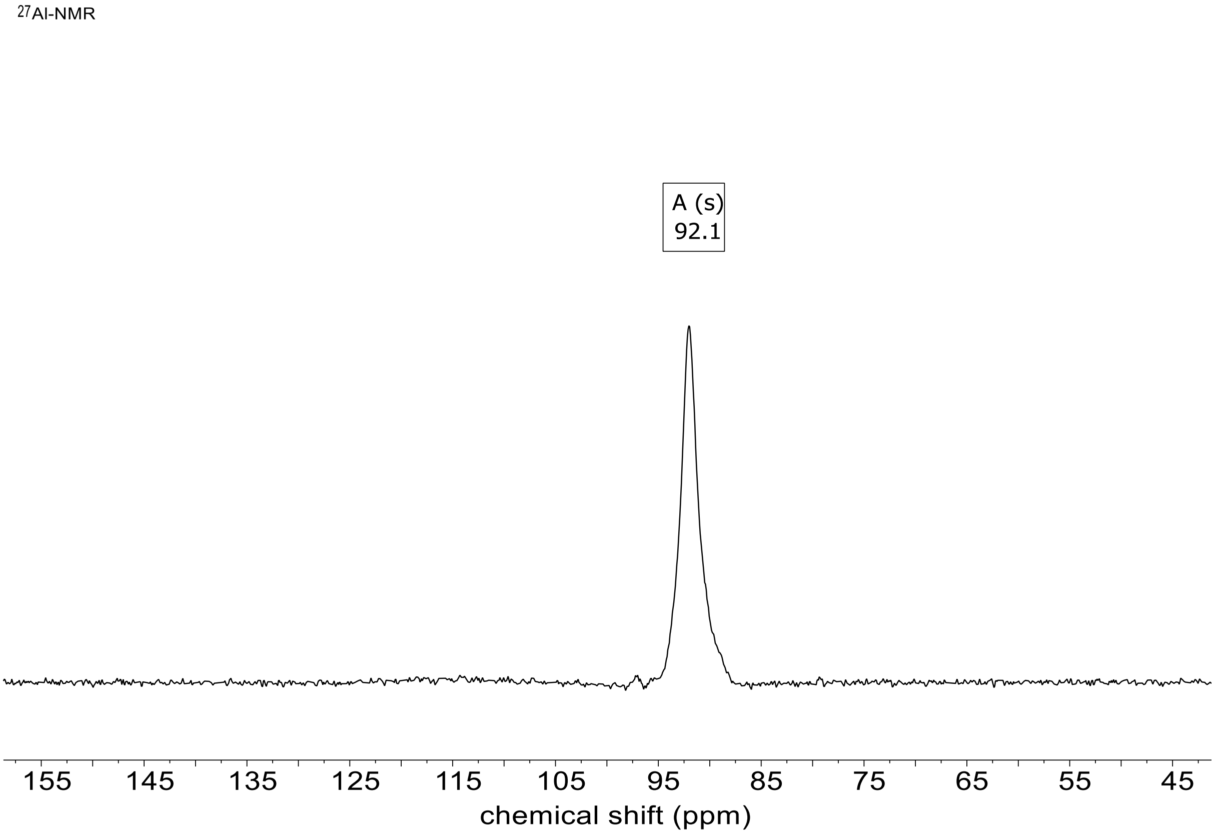

$^{31}P\{^1H\}$-NMR-spectrum of **6**:

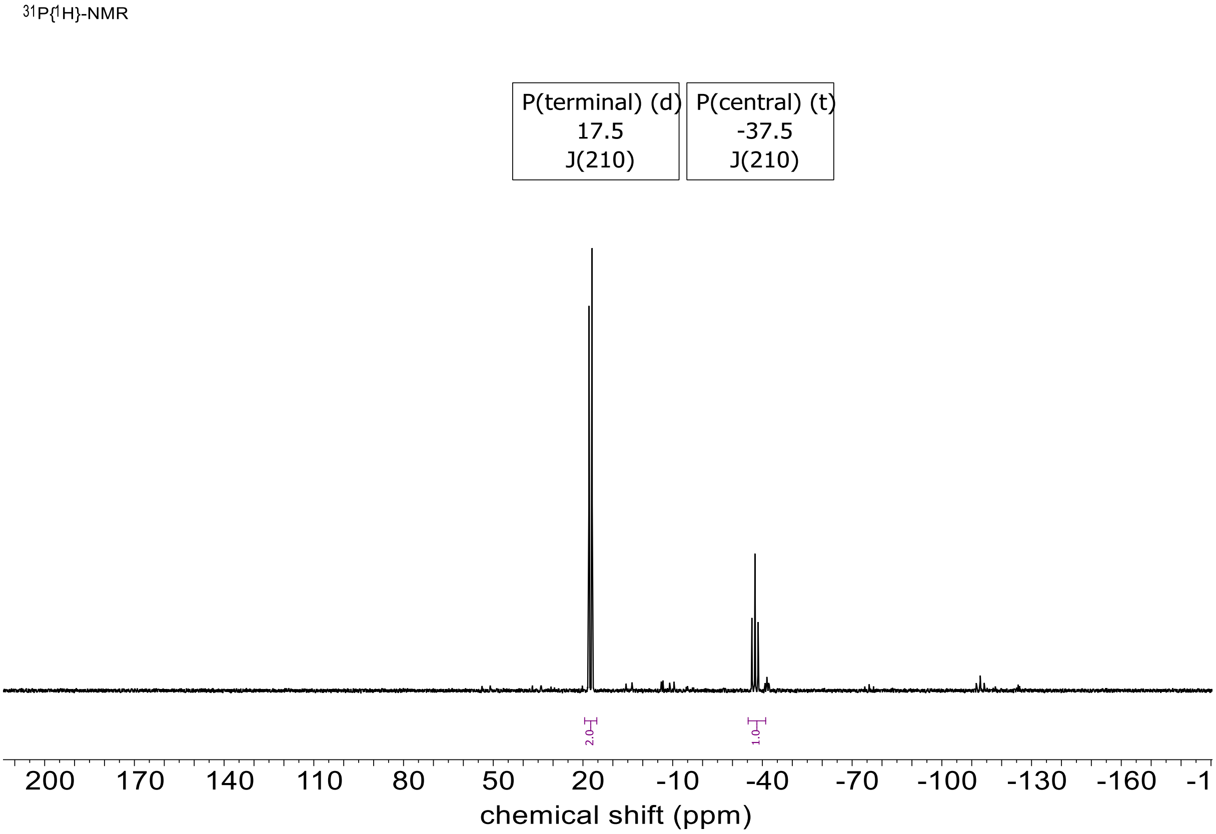

NMR-spectra of **7**:

^{1}H-NMR

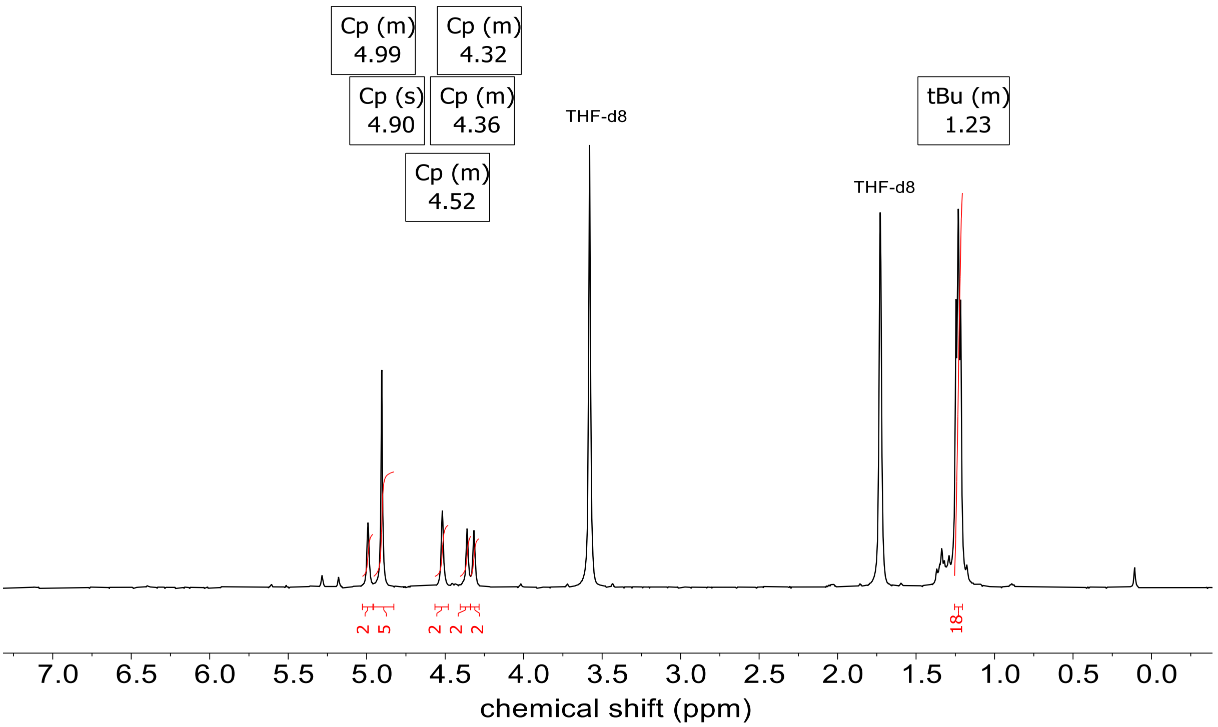

^{13}C-NMR

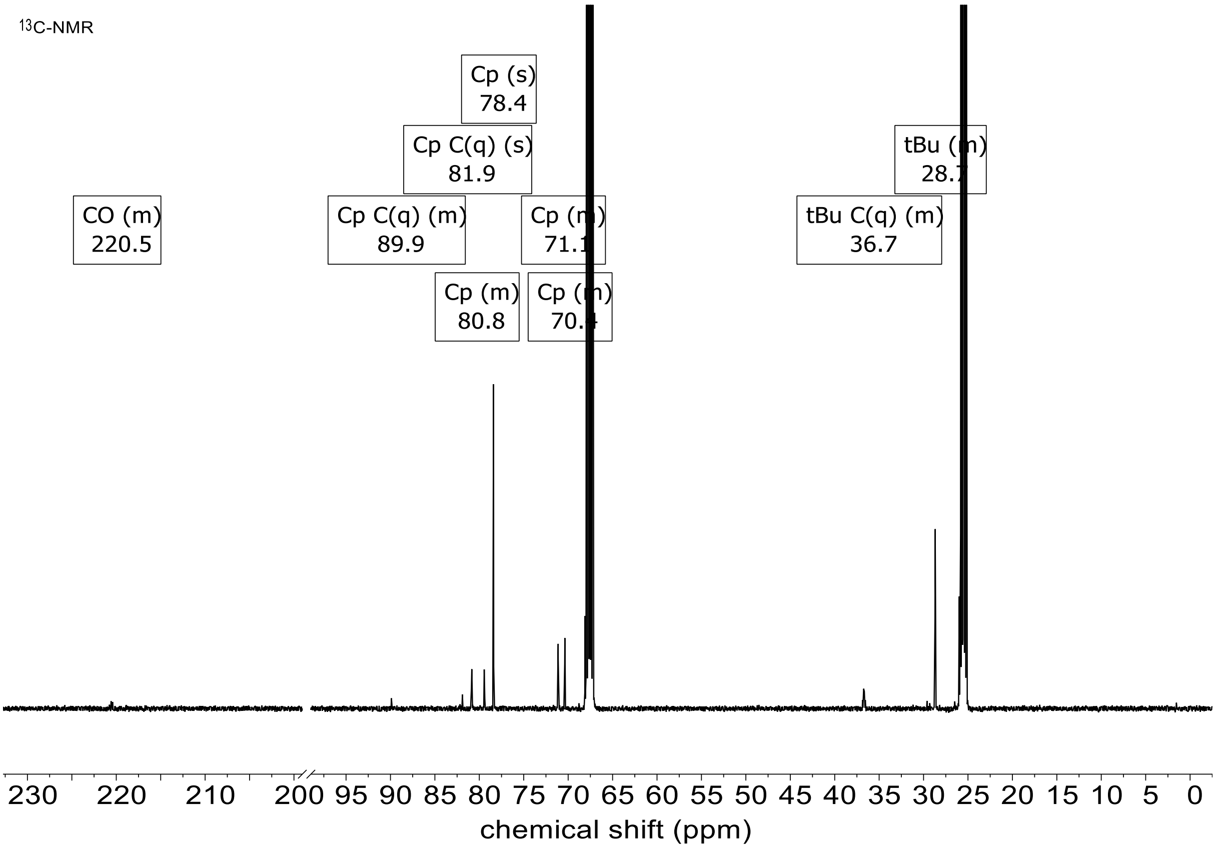

$^{31}P\{^{1}H\}$-NMR

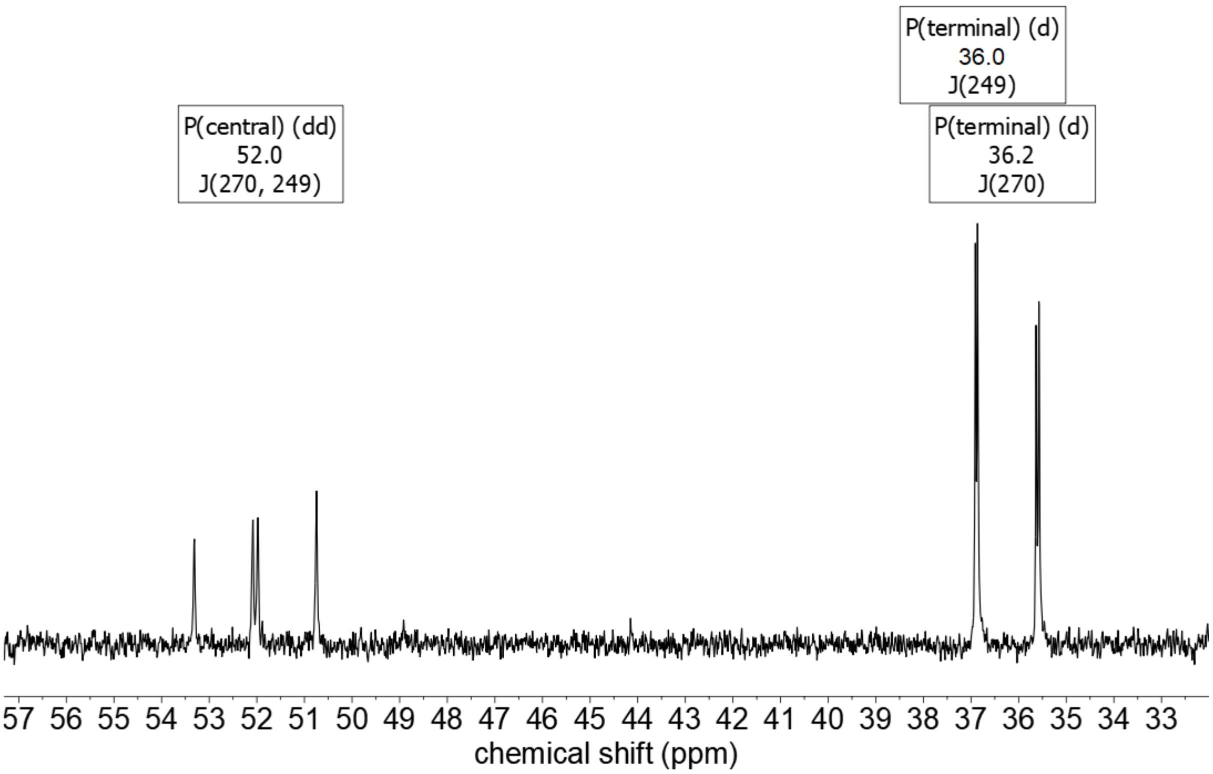

NMR-spectra of **8**:

^{1}H-NMR

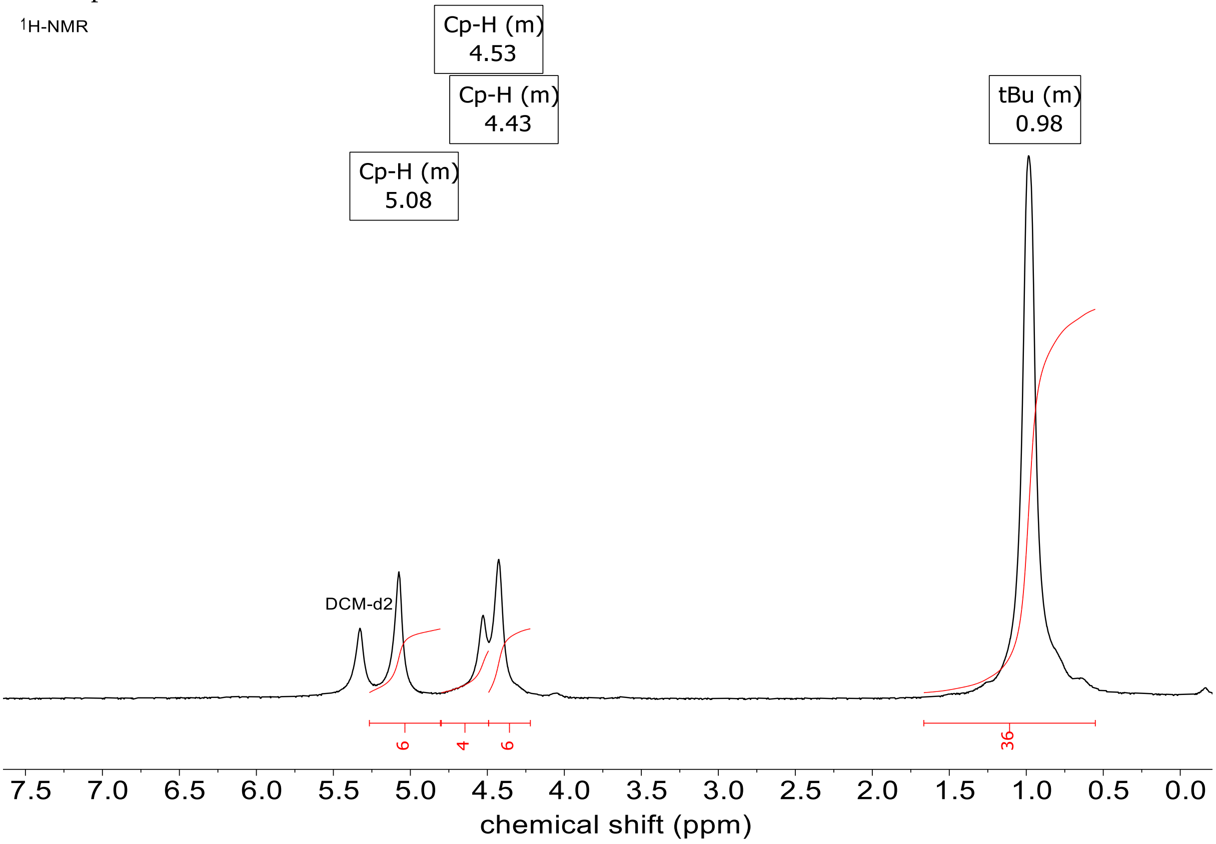

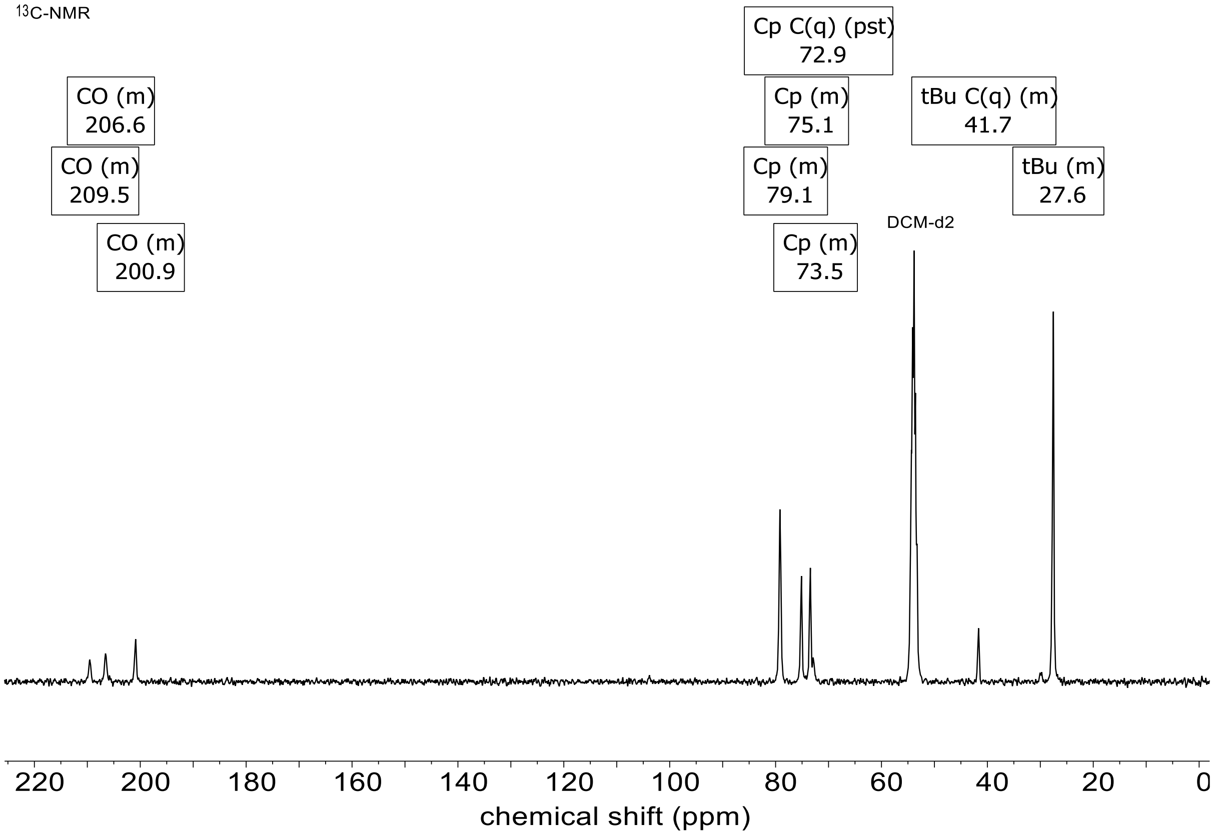
13C-NMR
Cp C(q) (pst)
72.9
CO (m)
206.6
Cp (m)
75.1
tBu C(q) (m)
41.7
CO (m)
209.5
Cp (m)
79.1
tBu (m)
27.6
DCM-d2
CO (m)
200.9
Cp (m)
73.5
220 200 180 160 140 120 100 80 60 40 20 0
chemical shift (ppm)

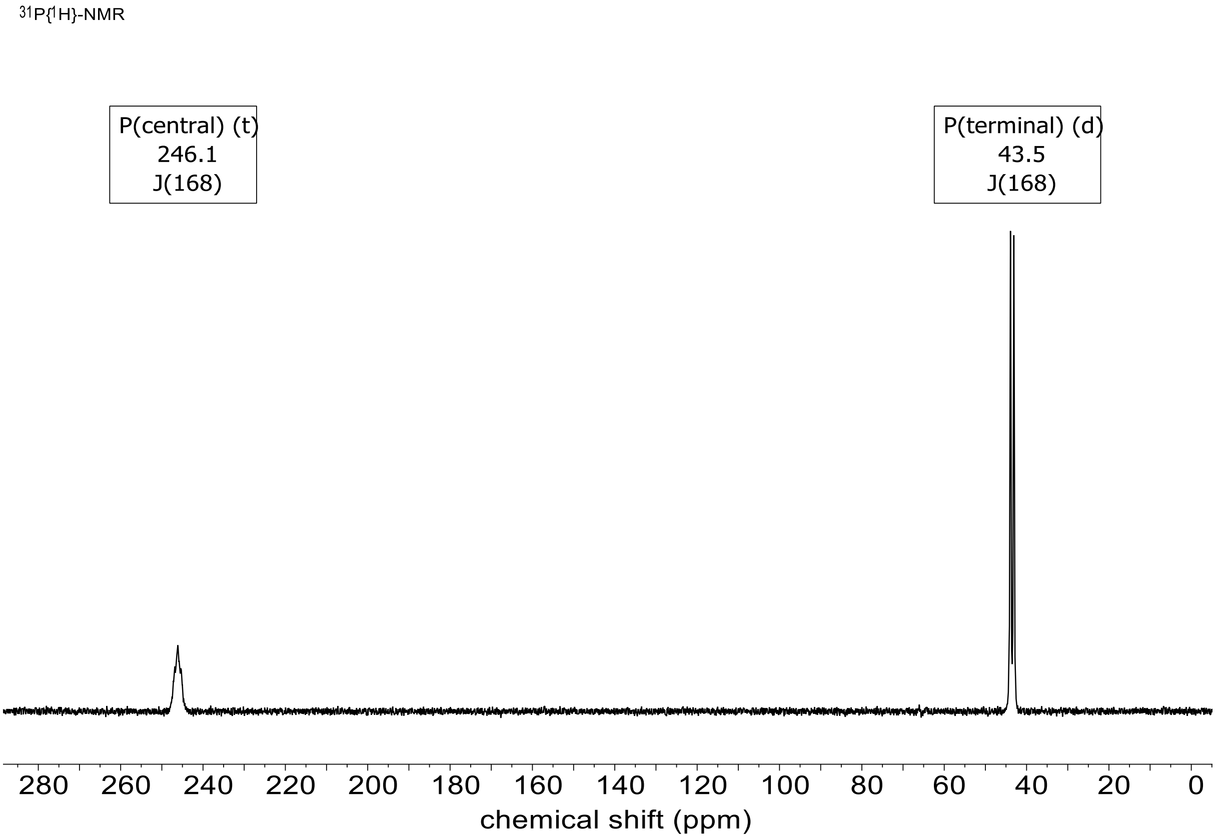
31P{1H}-NMR
P(central) (t)
246.1
J(168)
P(terminal) (d)
43.5
J(168)
280 260 240 220 200 180 160 140 120 100 80 60 40 20 0
chemical shift (ppm)

References

[1] Borucki, S.; Kelemen, Z.; Maurer, M.; Bruhn, C.; Nyulaszi, L.; Pietschnig, R. *Chem. Eur. J.* **2017**, *23* (43), 10438-10450.
[2] Edgell, W. F.; Lyford, J. *Inorg. Chem.* **1970**, *9* (8), 1932-1933.
[3] Gladysz, J. A.; Williams, G. M.; Tam, W.; Johnson, D. L.; Parker, D. W.; Selover, J. C. *Inorg. Chem.* **1979**, *18* (3), 553-558.
[4] Sheldrick, G. *Acta. Crystallogr. C.* **2015**, *71* (1), 3-8.
[5] Dolomanov, O. V.; Bourhis, L. J.; Gildea, R. J.; Howard, J. A. K.; Puschmann, H. *J. Appl. Cryst.* **2009**, *42* (2), 339-341.
[6] Macrae, C. F.; Edgington, P. R.; McCabe, P.; Pidcock, E.; Shields, G. P.; Taylor, R.; Towler, M.; van de Streek, J. *J. Appl. Crystallogr.* **2006**, *39* (3), 453-457.
[7] M. J. Frisch, G. W. Trucks, H. B. Schlegel, G. E. Scuseria, M. A. Robb, J. R. Cheeseman, G. Scalmani, V. Barone, G. A. Petersson, H. Nakatsuji, X. Li, M. Caricato, A. V. Marenich, J. Bloino, B. G. Janesko, R. Gomperts, B. Mennucci, H. P. Hratchian, J. V. Ortiz, A. F. Izmaylov, J. L. Sonnenberg, Williams, F. Ding, F. Lipparini, F. Egidi, J. Goings, B. Peng, A. Petrone, T. Henderson, D. Ranasinghe, V. G. Zakrzewski, J. Gao, N. Rega, G. Zheng, W. Liang, M. Hada, M. Ehara, K. Toyota, R. Fukuda, J. Hasegawa, M. Ishida, T. Nakajima, Y. Honda, O. Kitao, H. Nakai, T. Vreven, K. Throssell, J. A. Montgomery Jr., J. E. Peralta, F. Ogliaro, M. J. Bearpark, J. J. Heyd, E. N. Brothers, K. N. Kudin, V. N. Staroverov, T. A. Keith, R. Kobayashi, J. Normand, K. Raghavachari, A. P. Rendell, J. C. Burant, S. S. Iyengar, J. Tomasi, M. Cossi, J. M. Millam, M. Klene, C. Adamo, R. Cammi, J. W. Ochterski, R. L. Martin, K. Morokuma, O. Farkas, J. B. Foresman and D. J. Fox, Gaussian 16 Rev. C.01, Wallingford, CT, **2016**. 12.
[8] a) S. Weller, S. H. Schlindwein, C. M. Feil, Z. Kelemen, D. Buzsáki, L. Nyulászi, S. Isenberg, R. Pietschnig, M. Nieger and D. Gudat, A, *Organometallics*, **2019**, *38*, 4717–4725. b) S. Dey, F. Roesler, C. Bruhn, Z. Kelemen, R. Pietschnig *Inorg. Chem. Front.*, **2023**, *10*, 3828–3843
[9] T. Lu and F. Chen, Multiwfn: A multifunctional wavefunction analyzer, *J. Comput. Chem.*, **2012**, *33*, 580-592.
[10] A. T. B. Gilbert, IQmol molecular viewer. Available at: http://iqmol.org (Accessed October, 2012).

Author Contributions

The manuscript was written through contributions of all authors. All authors have given approval to the final version of the manuscript.
